# Astragali radix (Huangqi): a time-honored nourishing herbal medicine

**DOI:** 10.1186/s13020-024-00977-z

**Published:** 2024-08-30

**Authors:** Yuyu Zhang, Zhejie Chen, Liping Chen, Qin Dong, Dong-Hua Yang, Qi Zhang, Jing Zeng, Yang Wang, Xiao Liu, Yuan Cui, Minglong Li, Xiao Luo, Chongjian Zhou, Mingzhu Ye, Ling Li, Yuxin He

**Affiliations:** 1 School of Food and Bioengineering, Xihua University, Chengdu, 610039 China; 2 Institute of Molecular Medicine (IMM), Shanghai Key Laboratory for Nucleic Acid Chemistry and Nanomedicine, Renji Hospital, School of Medicine, Shanghai Jiao Tong University, Shanghai, 200127 China; 3 School of Comprehensive Health Management, Xihua University, Chengdu, 610039 China; 4 New York College of Traditional Chinese Medicine, Mineola, NY 11501 USA; 5 Pengzhou Hospital of Traditional Chinese Medicine, Pengzhou, 611930 China; 6 Chengdu Institute for Drug Control, NMPA Key Laboratory for Quality Monitoring and Evaluation of Traditional Chinese Medicine, Chengdu, 610045 China; 7 HuBei Guizhenyuan Chinese Herbal Medicine Co.Ltd., Hong’an, 438400 China; 8 Chengdu University of Traditional Chinese Medicine, Chengdu, 611137 Sichuan China

**Keywords:** *Astragalus membranaceus*, Huangqi, Natural products, Ethnobotany, Ethnopharmacological uses

## Abstract

Astragali radix (AR, namded Huangqi in Chinese) is the dried root of *Astragalus membranaceus* (Fisch.) Bge. var. *mongholicus* (Bge.) Hsiao or *Astragalus membranaceus* (Fisch.) Bge. As a widely used ethnomedicine, the biological activities of AR include immunomodulatory, anti-hyperglycemic, anti-oxidant, anti-aging, anti-inflammatory, anti-viral, anti-tumor, cardioprotective, and anti-diabetic effects, with minimum side effects. Currently, it is known that polysaccharides, saponins, and flavonoids are the indispensable components of AR. In this review, we will elaborate the research advancements of AR on ethnobotany, ethnopharmacological practices, phytochemicals, pharmacological activities, clinical uses, quality control, production developments, and toxicology. The information is expected to assist clinicians and scientists in developing useful therapeutic medicines with minimal systemic side effects.

## Introduction

The genus Astragalus is a group of Leguminosae, which has an indistinguishable species composition. To date, nearly 2000 species of Astragalus have a very wide distribution in northern temperate zones. In Europe, there are about 130 Astragalus species [1]. Worldwide, common names of astragalus plants include milkvetch (most species), goat's-thorn, and locoweed (some species in North America). In China, Astragalus is not only the common name of a well-known herb medicine, but also the name of the plant group from which it originates. Astragali radix is called "Huangqi” in China. Based on the 2020 edition of Chinese Pharmacopoeia issued by the Chinese government, both the dried root of the *Astragalus membranaceus* (Fisch.) Bge. var. *mongholicus* (Bge.) Hsiao and *Astragalus membranaceus* (Fisch.) Bge. are utilized as “Huangqi” in the medical practice of Traditional Chinese Medicine (TCM). AR was included for the first time as a high-quality tonic in the classic work named Shennong’s Classic of Materia Medica (神農本草經) [2], and has been applied in TCM over 2000 years. According to the theoretical system of TCM, this herbal medicine has a warm nature, sweet in flavor, and acts on the spleen and lung. Generally, after being cultivated for more than 3 years, AR is harvested in spring or autumn [3]. AR is mainly produced in Neimenggu, Shanxi, Gansu, and Heilongjiang provinces in China. Clinical practitioners of TCM believe that AR can lift yang and tonify Qi, reduce swelling, diffuse water, relieve sweat, fix the surface, generate muscle, and promote wound healing [4]. Therefore, AR is utilized as a time-honored tonic, which can be applied to clinical interventions of sub-health people, weak elderly, and the chronic disease patients with unsound immunity. Besides, AR is also a high-value raw material for the development of healthy foods in China, for the positive regulatory effects on regulate immunity, delay senescence, and resist fatigue [5]. At present, researchers have demonstrated that AR has clinical drug and nutraceutical applications potentials in immunomodulatory, anti-hyperglycemic, anti-oxidant, antiaging, anti-inflammatory, anti-viral, anti-tumor, cardioprotective, and anti-diabetic effects. Besides, it is also revealed that these biological activities are accompanied with minimum toxic and side effects.

Currently, over 200 small molecule chemical substances, containing saponins and flavonoids, have been isolated and identified from AR. Notably, an increasing number of research have proved that polysaccharides are also an indispensable active ingredient of AR [2, 4, 5]. About 40 kinds of triterpenoid saponins have been identified, including saponins, isoflavone saponins, acetyl saponins, and soybean saponins. Besides, there are more than 30 kinds of flavonoids, composed of flavonoids, formononetin, and its glycosides. In addition, AR polysaccharides are mainly composed of dextran and heteropolysaccharides [6–8]. Most of the heteropolysaccharides are water-soluble acidic heteropolysaccharides, primarily composed of glucose, galactose, rhamnose and arabinose [2]. In this review article, we will comprehensively introduce the active ingredients and mechanisms of action, clinical applications, quality control, and safety evaluation of AR. This study will provide rationales for development and utilization of AR in clinical drugs and nutraceuticals.

## Ethnobotany

**Fig. 1:**
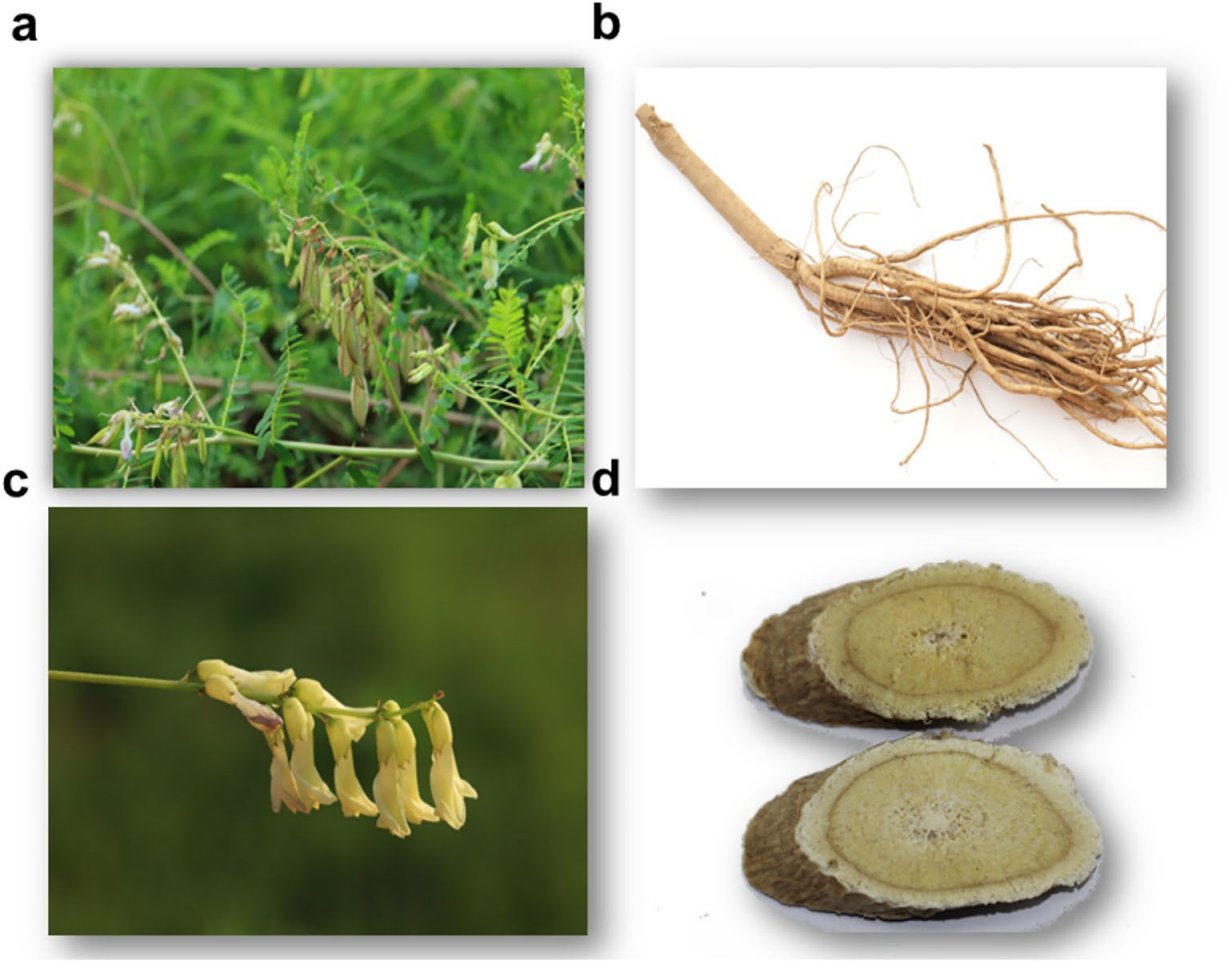
Plant overview (**a**), dry roots (**b**), racemes (**c**), and slices (**d**) of *Astragalus membranaceus* (Fisch.) Bge. var. *mongholicus* (Bge.) Hsiao

*Astragalus membranaceus* (Fisch.) Bge. is an herb with a height of 50–100 cm. The main roots are thick, woody, branched, and gray–white. Stems are erect, upper branched, finely angled, and have white villi. Pinnate compound leaves have 13–27 leaflets that are 5–10 cm long; petioles are 0.5–1 cm long; stipules are detachment, ovate-lanceolate or linear-lanceolate, 4–10 cm long, with white pubescence or subglabrous underneath; Leaflets are ovate or oblong-ovate, 7–30 cm long and 3–12 cm wide, apex obtuse-rounded or slightly concave, with small pointed or obscure, base rounded, nearly glabrous, underlying by white villi. Racemes are slightly dense, with 10–20 flowers; pedicels are nearly equal or longer compare with leaves, and significantly elongated during fruit stage; bracts are linear lanceolate, 2–5 cm long, with white pubescence on the back; Corolla is yellow or yellowish, 12–20 mm long, apex concave, the base with short petiole, wing petal shorter than flag petal. The petal is oblong, base with short ears, and the petiole is longer than petal about 1.5 times, keel petal and wing petal are nearly equal length. The pod is thin, slightly inflated, semi-elliptic, 20–30 mm long, 8–12 mm wide, apex spiny, with white or black pubescent on both sides; seeds are 3–8 mm. The flowering season is open from June to August and the fruit period is from July to September (Fig. 1). Besides, *Astragalus membranaceus* Bge. var. mongholicus (Bge.) Hsiao is shorter than *Astragalus membranaceus* (Fisch.) Bge, with smaller leaflets, 5–10 mm long, 3–5 mm wide, and glabrous pods.

**Fig. 2:**
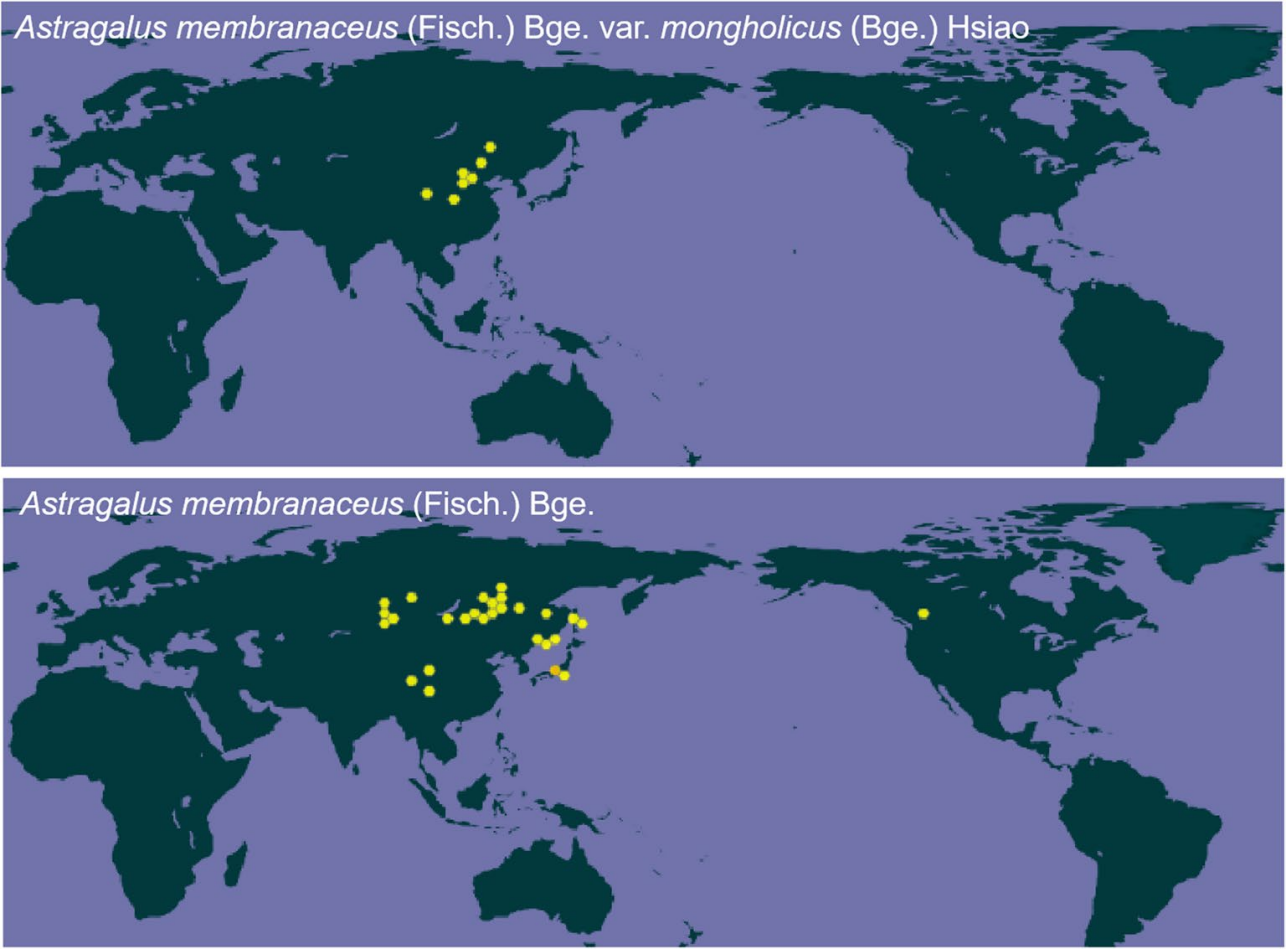
The global distribution of medicinal resources of AR. Data from the Global Biodiversity Information Facility

Researchers used ArcGIS to investigate its appropriate cultivating environment in China. The appropriate production areas for this herb are usually in mountainous regions with relatively little precipitation, including Gansu, Heilongjiang, Inner Mongolia and Ningxia, covering an area of about 506,000 square kilometers [9]. Besides, Data from the Global Biodiversity Information Facility showed that the medicinal resources of AR on a global scale are mainly distributed in the northern regions of China, as well as neighboring regions of Russia and northern China (Fig. 2). The wild medicinal resources of AR have declined drastically because of massive and prolonged excavation. The *Astragalus membranaceus* (Fisch.) Bge. and *Astragalus membranaceus* (Fisch.) Bge. var. *mongholicus* (Bge.) Hsiao or *Astragalus membranaceus* (Fisch.) Bge. are in danger of extinction. The wild medicinal resources of AR in China have been officially classified as third-class national protected plants by the government, and the AR currently used for clinical purposes is exclusively produced via artificial means.

## Ethnopharmacological practices

AR was used as a medicinal plant for more than 2000 years. Formulas using AR as the main ingredient in “Treatise on Miscellaneous Diseases of Typhoid Fever” written by Zhang Zhongjing (the “medical sage” in TCM) at the end of the Eastern Han Dynasty, including Fangji Huangqi Tang, Huangqi Jianzhong Tang, Wutou Tang, Huangqi Guizhi Wuwu Tang, and Huangqi Gui Zhi Shaoyao Tang. AR has been documented in other well-known TCM manuals, and these ancient texts describe the efficacy of AR and the herbs used in combination. In this paper, the prescriptions of AR on immunomodulation recorded in ancient formulas are summarized (Table 1). AR in other classical prescriptions related to immunomodulation have been documented in other medical works. Besides, AR has been traditionally used as analgesic, anticancer, antidiarrheal, anti-infective, anti-inflammatory, anti-fatigue, tonic, and hypoglycemic agents in in clinical practice of TCM [10].

**Table 1:** Main application of AR in classical prescriptions related to immune regulation

Prescription names	Main Materials	Traditional Indications	Provenances
Buyi huangqi pill (*補益黃芪丸*)	Astragali Radix Poria Alba Atractylodis Macrocephalae Rhizoma	Used for Lack of energy, dizziness, weakness of waist and knee, cold limbs	“Taiping Shenghui Fang” (*太平聖惠方*)
Buzhong Yiqi Decoction (*補中益氣湯*)	Astragali Radix Atractylodis Rhizoma Citri Reticulatae Pericarpium	Used for poor appetite, tired limbs, spontaneous sweating, prolonged diarrhea and persistent dysentery	“Neiwaishang Bianhuo Lun” (*內外傷辨惑論*)
Huangqi jianzhong Decoction (*黃芪建中湯*)	Astragali Radix Cinnamomi Ramulus Zingiberis Rhizoma Recens	Used for Long illness leads to weakness, Overwork, Weak pulse	“Synopsis of the Golden Chamber” (*金匱要略*)
Fangji Huangqi Decoction (*防己黃芪湯*)	Stephaniae Tetrandrae Radix Astragali Radix Glycyrrhizae Radix et Rhizoma Atractylodis Macrocephalae Rhizoma	Used for chronic glomerulonephritis, cardiogenic edema, rheumatoid arthritis	“Synopsis of the Golden Chamber” (*金匱要略*)
Yuye Decoction (*玉液湯*)	Dioscoreae Rhizoma Astragali Radix Anemarrhenae Rhizoma Pueraria Lobate Radix	Used for after radiotherapy, diabetes mellitus, hyperthyroidism, children's summer fever, diabetes insipidus and other thirsty urine mostly belong to deficiency of both spleen and kidney	“Records of Traditional Chinese and Western Medicine in Combination” (*醫學衷中參西錄*)
Huangqi buwei Decoction (*黃芪補胃湯*)	Astragali Radix Angelicae Sinensis Radix Cinnamomi Cortex	Used for pregnant women suffering from deficiency of Qi and cold, fear of cold and abdominal pain, thus abortion	“Bianzheng Lv” (*辨證論*)
Buyang Huanwu Decoction (*補陽還五湯*)	Astragali Radix Angelicae Sinensis Radix Chuanxiong Rhizome Paeoniae Radix Rubra	Used for Sequelae of cerebrovascular accident, coronary heart disease, poliomyelitis	“Yilin Gaicuo” (*醫林改錯*)
Zaizao San (*再造散*)	Ginseng Radix et Rhizoma Astragali Radix Glycyrrhizae Radix et Rhizoma	Used for deficiency of Yang Qi, tiredness and sleepiness, cold limbs, pale complexion, exogenous cold	“Shanghan Liushu” (*傷寒六書*)
Yupingfeng San (*玉屏風散*)	Saposhnikoviae Radix Astragali Radix Atractylodis Macrocephalae Rhizoma	Used for Anaphylactic rhinitis, upper respiratory tract infection, glomerulonephritis are prone to recurrent cold	“Jiuyuan Fang” (*究原方*)
Danggui Buxue Decoction (*當歸補血湯*)	Astragali Radix Angelicae Sinensis Radix	Used for ulcer not healing, Fever and headache during menstruation and postpartum period	“Neiweishang Bianhuo Lun” (*內外傷辨惑論*)

## Phytochemicalsthnobotany

So far, researchers have isolated and identified over 200 compounds from AR, and some of these components have shown biological activity in vivo or in vitro. Understanding these various compounds of AR is helpful for carrying out scientific research, clinical applications and understanding their biological activity mechanisms. Therefore, 117 compounds with biological activities have been reported in this paper (Tables 2, 3, and 4), including polysaccharides (1–25), Triterpenoid saponins (26–55), and flavonoids (56–117).

**Table 2:** Polysaccharides isolated and purified from AR

NO	Name	Molecular mass (Da)	Monosaccharide composition	Molar ratio	Refs.
1	APS-A1	2.62 × 10^6^	1,4,6-*α*-D-Glcp		[11]
2	APS-B1	4.95 × 10^6^	glucose, galactose and arabinose	75.24.17.27:19.35	[11]
3	APS2-I	1 .96 × 10^6^	Man, Rha, GlcA, GalA, Glc, Gal, Xyl, and Ara	2.3:4.8:1.7:14.0:5.8:11.7:2.8:12.6	[12]
4	APS3-I	3.91 × 10^6^	Rha, GalA, Glc, Gal, and Ara	0.8:2.3:0.8:2.3:4.1	[12]
5	APS-I	3.84 × 10^4^	D-Galactose, D-Glucose	1:49.76	[14]
6	APS-I	2 × 10^5^	L-Rhamnose,D-Galacturonic acid, D-Galactose, D-Glucose,L-Arabinose	0.1:0.39:13.4:17.2:1	[15]
7	APS-II	1 × 10^4^	L-Rhamnose, D-Galacturonic acid, D-Galactose, D-Glucose, L-Arabinose	0.14:0.14:9.6:24.04:1	[15]
8	APS-II	5.2 × 10^3^	L-Rhamnose, D-Galactose, D-Glucose	1:2.99:16.26	[14]
9	APS-III	3.0 × 10^2^	L-Rhamnose, D-Galacturonic acid, D-Galactose, D-Glucose, L-Arabinose	0.375:0.375:18.8:90.5:1	[15]
10	APS-1	2.57 × 10^5^	D-Glucose		[16]
11	APS-2	4.01 × 10^4^	L-Arabinose		[16]
12	APS-3	1.53 × 10^4^	L-Rhamnose, D-Glucose, D-Galactose, L-Arabinose	1:10.76:6.55:12	[16]
13	APS-4	3.2 × 10^3^	D-Galactose, L-Arabinose	3.02:1	[16]
14	LM_W_-ASP	5.6 × 10^3^	Glc, Gal, Ara, Xyl, and GalA	10.0:1.3:1.7:1.0:0.9	[17]
15	APS-I	1.06 × 10^4^	D-Mannose, L-Rhamnose, D-Glucuronic acid, D-Galacturonic acid, D-Glucose, D-Galactose	29.12:1.89:4.00:1.35:1:81.97	[18]
16	APS-II	2.47 × 10^6^	D-Mannose, L-Rhamnose, D-Glucuronic acid, D-Galacturonic acid, D-Glucose, D-Galactose, D-Xylose	50.46:1.16:1:2.27:2.66:15.72:7.86	[18]
17	ASP-I	4.32 × 10^4^	D-Galactose, D-Glucose, L-Arabinose	1.0:24.8:2.5	[19]
18	ASP-II	2.81 × 10^5^	L-Rhamnose, D-Galacturonic acid, D-Glucose, D-Galactose, L-Arabinose	1.2:1.0:19.3:2.5:8.7	[19]
19	MAPS-5	1.32 × 10^4^	*α*-D-(1–4) Glucos		[20]
20	APS	2 × 10^6^	D-Mannose, D-Glucose, D-Xylose, L-Arabinose, D-Glucuronic acid, L-Rhamnose	0.27:12.83:1.63:0.71:1.04:0.56	[21]
21	APS	2.7 × 10^4^	*α*-D-glc		[22]
22	APS	3.01 × 10^5^	L-rhamnose, D-xylose, D-glucose	1:4:5:1.5	[23]
23	APS	2.1 × 10^3^	arabinose, galactose, glucose, and xylose	1.00:0.98:3.01:1.52	[24]
24	cAMPs-1A	1.2 × 10^4^	fucose, arabinose, galactose, glucose, and xylose	0.01:0.06:0.20:1.00:0.06	[13]
25	AAP-2A	2.252 × 10^3^	L-Rhamnose, D-Galactose, L-Arabinose, D-Glucose	1:2.13:3.22:6.18	[25]

**Table 3:** Triterpenoid saponins isolated from AR

NO	Name	M.F	M.W	Refs.
26	Astragaloside IV	C_41_H_68_O_14_	784	[26]
27	Astragaloside III	C_41_H_68_O_14_	784	[26]
28	Isoastragaloside II	C_43_H_70_O_15_	826	[26]
29	Astragaloside I	C_45_H_72_O_16_	868	[26]
30	Isoastragaloside I	C_45_H_72_O_16_	868	[26]
31	Acetylastragaloside I	C_47_H_74_O_17_	910	[26]
32	Isoastragaloside IV	C_41_H_68_O_14_	784	[27]
33	Astragaloside VII	C_47_H_78_O_19_	946	[26]
34	Brachyoside B	C_36_H_60_H_10_	65	[28]
35	Astragaloside II	C_43_H_70_O_15_	826	[26]
36	Astragaloside VI	C_47_H_78_O_19_	946	[27]
37	Astragaloside V	C_47_H_78_O_19_	946	[26]
38	Cyclocephaloside II	C_43_H_70_O_15_	826	[29]
39	Astramembranoside A	C_42_H_70_O_15_	814	[30]
40	Agroastragaloside IV	C_49_H_80_O_20_	988	[31]
41	Agroastragaloside III	C_51_H_82_O_21_	1030	[31]
42	Cycloaraloside A	C_36_H_60_O_10_	652	[28]
43	Astramembrannin II	C_35_H_58_O_9_	622	[30]
44	Cyclocanthoside A	C_35_H_60_O_9_	624	[27]
45	Astramembranoside B	C_41_H_70_O_14_	786	[30]
46	Agroastragaloside II	C_43_H_72_O_15_	828	[30]
47	Cyclocanthoside E	C_41_H_70_O_14_	786	[30]
48	Agroastragaloside I	C_45_H_74_O_16_	870	[30]
49	Agroastragaloside V	C_43_H_72_O_14_	812	[32]
50	Astragaloside VIII	C_47_H_76_O_17_	912	[26]
51	Soyasaponin I	C_48_H_78_O_18_	942	[26]
52	Soyasapogenol B	C_30_H_50_O_3_	458	[33]
53	Azukisaponin V methylester	C_49_H_80_O_18_	956	[30]
54	Lupeol	C_30_H_50_O	426	[34]
55	Ursolic acid	C_30_H_48_O_3_	456	[32]

**Table 4:** Flavonoids isolated from AR

NO	Name	M.F	M.W	Refs
56	4ʹ,7-dihydroxyflavone	C_15_H_10_O_4_	254	[28]
57	3ʹ,4ʹ,7-trihydroxyflavone	C_15_H_10_O_5_	270	[28]
58	Oroxylin-A	C_16_H_12_O_5_	284	[38]
59	Wogonin	C_16_H_12_O_5_	284	[38]
60	( −)-Liquiritigenin	C_15_H_12_O_4_	256	[28]
61	4ʹ-hydroxyflavanone 7‐O‐*β*-D-glucoside	C_21_H_22_O_9_	418	[39]
62	Quercetin	C_15_H_10_O_7_	302	[40]
63	Kaempferol	C_15_H_10_O_6_	286	[41]
64	Formononetin	C_16_H_12_O_4_	268	[28]
65	Formononetin 7-O‐*β*-D-glucoside	C_22_H_22_O_9_	430	[28]
66	6ʹʹ‐Acetyl‐ononin	C_24_H_24_O_10_	472	[32]
67	Calycosin	C_16_H_12_O_5_	284	[28]
68	Calycosin 7‐O‐*β*‐D‐glu	C_22_H_22_O_10_	446	[28]
69	Calycosin 7‐O‐*β*- D‐(6‐O‐acetly)‐glucopyranoside	C_24_H_24_O_11_	488	[29]
70	Genistein	C_15_H_10_O_5_	270	[41]
71	Genistin	C_21_H_20_O_10_	432	[40]
72	Pratensein	C_16_H_22_O_6_	300	[28]
73	Odoratin 7‐O‐*β*‐D‐glucopyranoside	C_23_H_24_O_11_	476	[41]
74	3ʹ‐methoxy‐5ʹ‐hydroxy‐isoflavone 7-O‐*β*-D‐glucoside	C_22_H_22_O_10_	446	[42]
75	3ʹ,7,8‐ trihydroxy‐4ʹ‐methoxyisoflavone	C_12_H_16_O_6_	300	[28]
76	8,3ʹ‐dihydroxy‐7,4ʹ‐dimethoxyisoflavone	C_17_H_14_O_6_	314	[43]
77	7,3ʹ-dihydroxy-8,4ʹ-dimethoxyisoflavone	C_17_H_14_O_6_	314	[43]
78	Calycosin 7‐O‐*β*-D-{6ʹʹ‐[(E)‐but‐2‐enoyl} ‐glucopyranoside	C_26_H_26_O_11_	514	[29]
79	Ammopiptanoside A	C_26_H_26_O_10_	498	[29]
80	3ʹ,7-Dihydroxy-5ʹ-methoxyisoflavone	C_16_H_12_O_5_	284	[42]
81	4ʹ,5,7-Trihydroxy-3ʹ-methoxyisoflavone	C_16_H_12_O_6_	300	[44]
82	Afrormosin	C_17_H_14_O_5_	298	[45]
83	Pratensein 7‐O‐*β*‐D‐glucopyranoside	C_22_H_22_O_11_	462	[46]
84	Sissotrin	C_22_H_22_O_10_	446	[44]
85	Calycosin 7‐O‐*β*‐D‐{6ʹʹ‐[(E)‐but‐2‐enoyl}‐galcopyranoside	C_26_H_26_O_11_	514	[47]
86	( −)-methylnissolin	C_17_H_16_O_5_	300	[29]
87	(6aR,11aR)-3,9,10-Tri-methoxypterocarpan	C_18_H_18_O_5_	314	[48]
88	( −)-Methylinissolin 3-O-*β*-D- glucoside	C_23_H_26_O_10_	462	[48]
89	( −)‐Methylinissolin 3‐O‐*β*‐D‐(6ʹ‐acetyl)‐glucoside	C_25_H_28_O_11_	504	[44]
90	(6aR,11aR)‐3,9‐Dimethoxy‐10‐hydroxypterocarpan	C_17_H_16_O_5_	300	[48]
91	Vesticarpan	C_16_H_14_O_5_	286	[29]
92	Licoagroside D	C_22_H_24_O_10_	448	[29]
93	( −)‐Methylinissolin 3‐O‐*β*‐D‐{6ʹ‐O‐[(E)‐but‐2‐enoyl]}‐glucoside	C_27_H_30_O_11_	530	[39]
94	Trifolinhizin	C_22_H_22_O_10_	446	[39]
95	Daidzein	C_15_H_12_O_4_	256	[49]
96	(3R)‐8,2ʹ‐Dihydroxy‐7,4ʹ‐dimethoxyisoflavan	C_17_H_18_O_5_	302	[43]
97	Isomucronulatol	C_17_H_18_O_5_	302	[44]
98	7‐O‐methylisomucronulatol	C_18_H_20_O_5_	316	[29]
99	Isomucronulatol‐7‐*β*‐O‐glucoside	C_23_H_28_O_10_	464	[48]
100	Isomucronulatol 7,2ʹ‐di‐O‐*β*‐glucoside	C_29_H_38_O_15_	626	[50]
101	(3R)‐7,2ʹ,3ʹ‐Trihydroxy‐4ʹ‐methoxy‐isoflavane	C_16_H_16_O_5_	288	[48]
102	(R)‐3‐(5‐Hydroxy‐2,3,4‐trimethoxyphenyl)‐chroman‐7‐ol	C_18_H_20_O_6_	332	[43]
103	Pendulone	C_17_H_16_O_6_	316	[29]
104	6ʹʹ‐O‐Acetyl‐(3R)‐2ʹ‐hydroxy‐3ʹ,4ʹ‐dimethoyl‐isoflavan 7‐O‐*β*‐glucopyranoside	C_25_H_30_O_11_	506	[44]
105	3ʹ‐Hydroxy‐2ʹ,4ʹ‐dimethoxyisoflavan	C_23_H_28_O_11_	480	[45]
	6‐O-*β*‐D-glucopyranoside			
106	Astraflavonoid C	C_23_H_28_O_11_	480	[41]
107	3,2ʹ‐Dihydroxyl‐3ʹ,4ʹ‐methoxyisoflavanone 7‐O‐*β*‐D‐glucoside	C_23_H_28_O_11_	480	[39]
108	(3R,4R)‐4,7‐Hydroxy‐2ʹ,3ʹ‐dimethoxyisoflavane 4ʹ‐O‐*β*-D-glucoside	C_23_H_28_O_11_	480	[39]
109	2ʹ,5ʹ‐Dicarbonyl‐3ʹ,4ʹ‐dimethoxyisoflavanequinone 7‐O‐*β*‐D‐glucoside	C_23_H_26_O_11_	478	[51]
110	hEchinatin	C_16_H_14_O_4_	270	[28]
111	Licochalcone B	C_16_H_14_O_5_	286	[28]
112	2ʹ,4,4ʹ-Trihydroxychalcone	C_15_H_12_O_4_	256	[28]
113	2ʹ-Methoxyisoliquiritigenin	C_16_H_14_O_4_	270	[28]
114	4,4ʹ,6ʹ-Trihydroxychalcone	C_15_H_12_O_4_	256	[39]
115	4-Methoxy-4ʹ,6ʹ-dihydroxychalcone	C_16_H_14_O_4_	270	[39]
116	4,4ʹ-Dimethyl-6ʹ-hydroxychalcone	C_17_H_16_O_2_	252	[39]
117	Sophorophenolone	C_16_H_10_O_6_	298	[50]

### Polysaccharides

AR polysaccharides (APS) are important active ingredients extracted from AR. APS is composed mainly of hetero-polysaccharides and glucans. Hetero-polysaccharides are mainly water-soluble acidic polysaccharides. Glucans include water-soluble *α*-(1–4)(1–6) glucans and water-insoluble *α*-(1–4) glucans. A recent study has identified two new polysaccharides in AR. APS-A1 **(1)** was a 1,4-*α*-D-Glcp backbone, and APS-B1 **(2)** was a heteropolysaccharide. The main chain of APS-B1 was made up of 1,4-*α*-d-glcp, 1,4,6-*α*-d-glcp, and 1,5-*α*-l-Araf, and the side chain consisted of 1,6-*α*-d-galp and T-*α*/*β*-Glcp [11]. Moreover, it was found that the two polysaccharides possessed anti-inflammatory effects and confirmed that the molecular weight, glycosidic bond, monosaccharide composition, and glyoxylate content of the polysaccharides might affect their anti-inflammatory activities. Another study also identified two polysaccharides, named APS2-I **(3)** and APS3-I **(4)** [12]. The researchers not only studied their structures in detail, but also confirmed that the two polysaccharides have good antioxidant and cardioprotective properties. Notably, the water-soluble heteropolysaccharide (cAMPs-1A) **(24)** can be obtained from AR in cold water (4 °C) [13]. In this paper, we provide a compendium of the APS that have been isolated and purified from AR (Table 2).

### Triterpenoid saponins

**Fig. 3:**
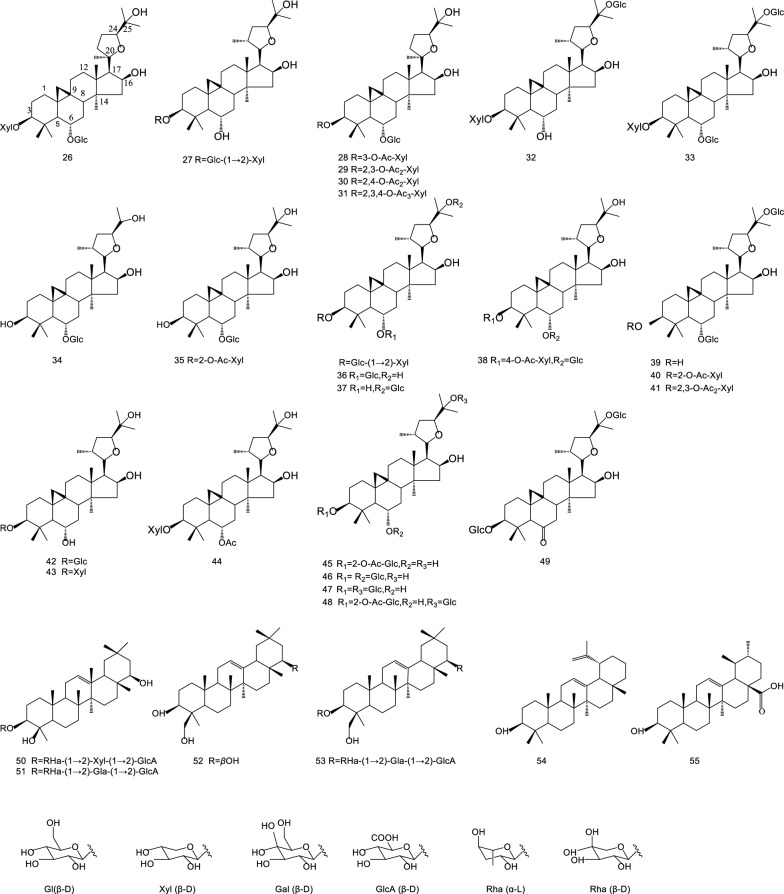
Chemical structures of triterpenoid saponins in AR

Triterpenoid saponins are characteristic bioactive compounds of AR, and are also the main medicinal ingredients of AR. There are about 40 types of triterpenoid saponins have been isolated and identified from AR (Table 3). Wherein, Astragalosides are important tetracyclic triterpenoid compounds, including Astragalosides I **(29)**, II **(35)**, III **(27)**, IV **(26)**, V **(37)**, VI **(36)**, VII **(33)**, and VIII **(50)**. They have a wide range of biological activities and significant pharmacological effects. Astragaloside is often used as a “labelled ingredient” in the Chinese Pharmacopoeia, and Astragaloside is also recorded in the European Pharmacopoeia and the British Pharmacopoeia. Besides, Chemical Structures of triterpenoid saponins extracted from AR are documented in Fig. 3.

### Flavonoids

**Fig. 4:**
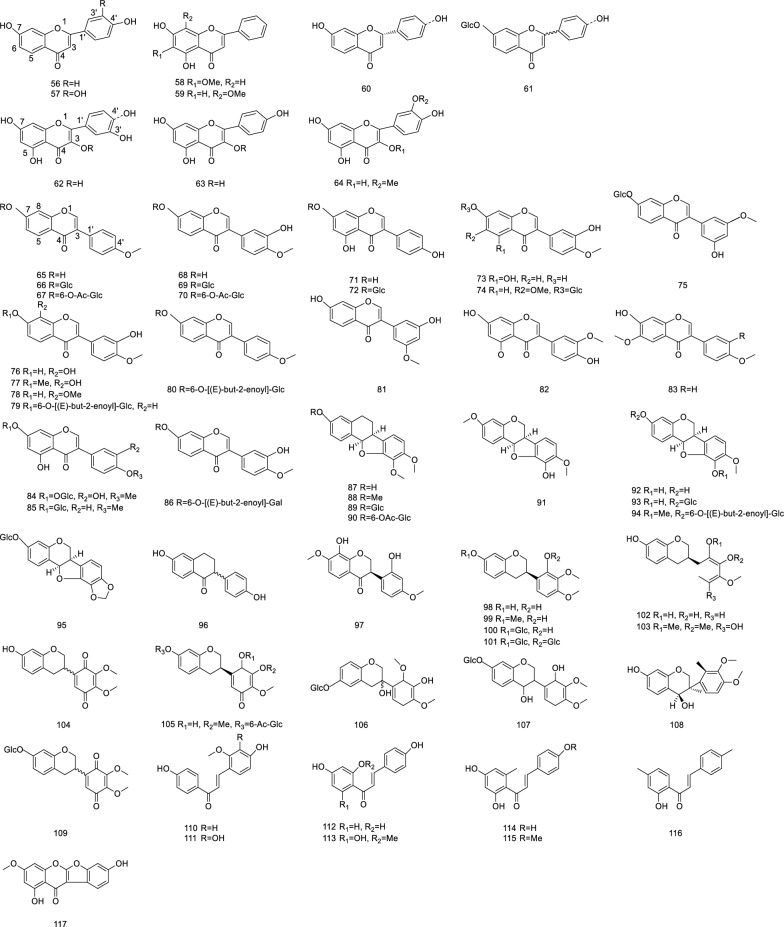
Chemical structures of flavonoids in AR

Flavonoids are the largest class of plant secondary metabolites, and compounds are based on a same C6-C3-C6 skeleton. According to the level of unsaturation and replace the pattern, it is possible to distinguish among flavonoids, flavanones, flavan-3-ols, anthocyanins, dihydroflavanols, and isoflavones [35]. Currently, isoflavones are widely studied by scholars. Calycosin-7-O-*β*-D-glucoside **(68)**, calycosin **(67)**, and formononetin **(64)** are the main isoflavone constituents of AR [36]. Owing to their complex structure, the bioavailability of flavonoids is very low, and a large number of flavonoids reach the gut in their prototypical form and interact with gut microbes to indirectly modulate various diseases [37]. Flavonoids extracted from AR are listed in Table 4 and their chemical structure are shown in Fig. 4.

### Others

In addition to the above types of chemical structure, AR is also rich in amino acid components, with high contents of aspartic acid, proline, arginine, aspartic acid, and alanine [52]. AR also contains phenylpropanoid compounds, organic acids, quinones, furans, inositols, and glycosides. In addition, AR contains overs 20 trace elements, including iron, manganese, cobalt, and copper [53].

## Pharmacological activities

**Fig. 5:**
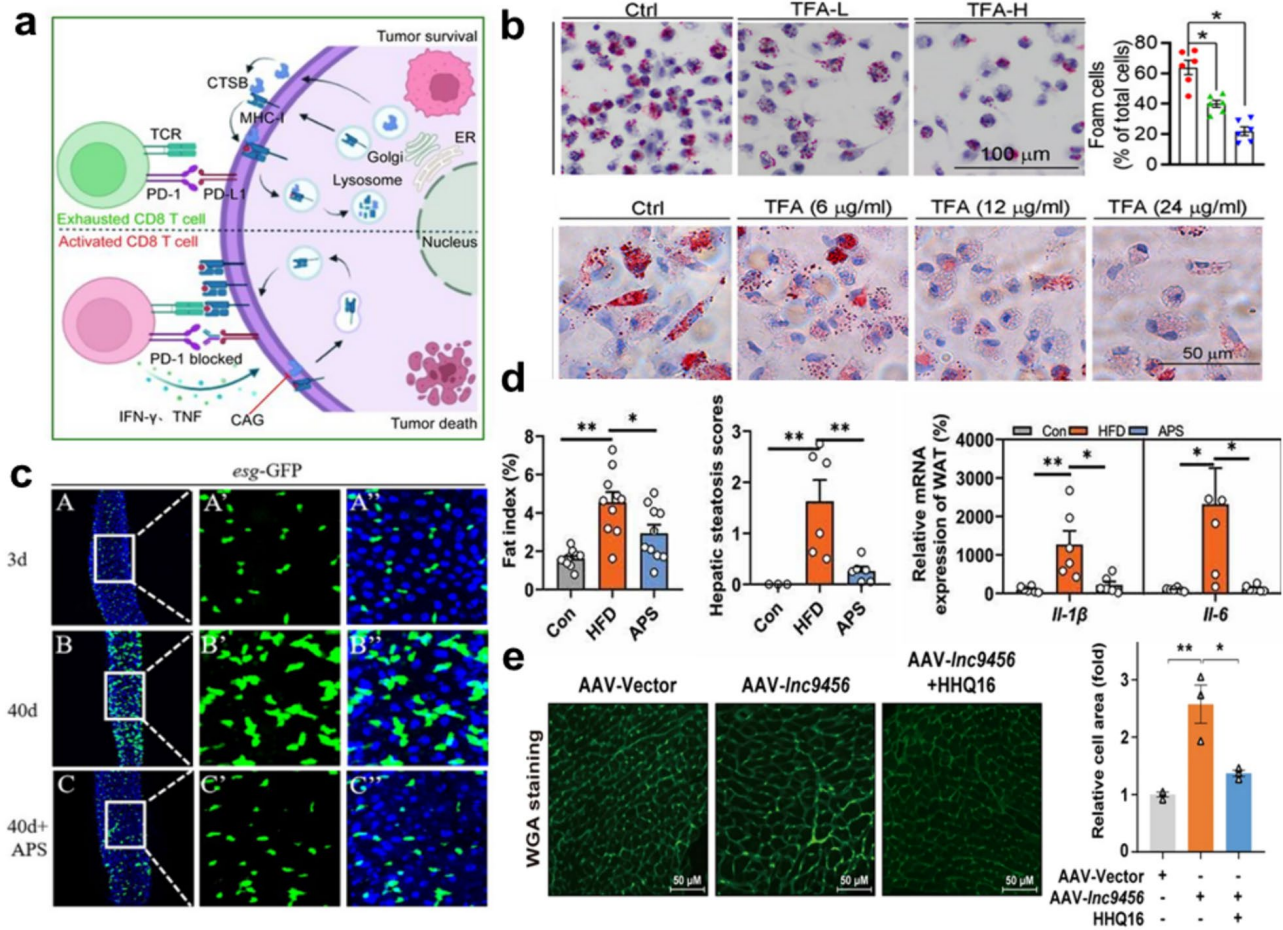
AR active ingredients exhibit a variety of actions.** a** Mechanisms of anti-tumour use of CAG in combination with PD-1 antibodies. This figure has been adapted/reproduced from ref [153] with permission from BMJ.** b** Inhibitory effect of TFA on foam cell and RAW264.7 cells formation using Oil Red O staining. This figure has been adapted/reproduced from ref [78] with permission from Frontiers.** c** Immunofluorescence images of the dissected midguts of esgGFP in 3-day flies, and 40-day-old flies fed with or without 3 mg/mL APS. This figure has been adapted/reproduced from ref [83] with permission from Elsevier.** d** Fat index, hepatic steatosis score, and relative expression of pro-inflammatory factors (IL-1*β* and IL-6) mRNAs in liver tissue (%) of mice fed on high-fat chow supplemented with 4% APS. This figure has been adapted/reproduced from ref. [154] with permission from Taylor & Francis.** e** Representative WGA staining of mouse myocardial tissue (left) and its quantification (right) in AAV-lnc9456 mice treated with HHQ16. This figure has been adapted/reproduced from ref. [155] with permission from Springer Nature

Modern pharmacological research demonstrated that AR has many pharmacological activities, including immunomodulatory activity, anti-tumor, anti-inflammation, anti-aging, and anti-heart failure. The rich biological activities of AR provided various possibilities for its clinical applications in TCM and pharmaceutical studies ensured its value (Fig. 5).

### Immunomodulatory activity

The immunomodulatory activity of AR has been in the spotlight for a long time. Literatures have demonstrated that the immunomodulatory properties of AR are based on its bioactive components, including APS, Astragalus flavonoids (AF), and Astragaloside (AS). Particularly, APS is a systemic immunomodulator linked to innate immunity, mucosal immunity, immune organs, and acquired immunity. A previous study found that AR is promising in the field of farmed animals, where it can greatly improve the immune organ index. The study showed that feeding mice with AR (5, 25 and 50 g/kg) for 30 days increased the splenic index of mice [54]. In a recent study, APS (0 and 2 g/kg) fed to crucian carp for 56 days was found to improve the immunological parameters of the spleen, kidney and liver of crucian carp [55]. Notably, research confirmed that APS promotes the growth of immune organs at a young age. According to reports, basal diets added with 0.2%, 0.4%, and 0.6% APS and fed to 1-day-old broiler chicks for 7 days significantly increased liver, pancreas, spleen, and bursa indices [56]. However, APS had no significant effect on immune organ indices in 14-day-old broilers. This observation was confirmed by another study in which 3 ml of Astragalus extract (ARE, 0.2 mg/L) was fed continuously to 7-day-old chickens for 6 weeks [57]. Except for APS, feeding total flavonoids of Astragalus (TFA) (25, 50, and 100 mg/kg) to mice for 7 days, significantly increased thymus and spleen indices [58]. The above findings demonstrated that the various bioactive ingredients of AR are effective in regulating immune organs. However, their related mechanisms are still lacking, and are being explored. Some scholars put forward that the APS modulated immune activity by modulating the gut microbiota [59]. Studies have shown that feeding APS to rats can improve the species and change the composition of the gut flora, including 12 key bacteria associated with weight gain and immune organ index [59]. Furthermore, another study indicated [60] that APS actuated the bursa toll-like receptor (TLR) 4 pathway via MyD88-independent pathway, leading to increased levels of SIgA content and chTLR4 mRNA expression/protein.

The mucosal immune system is the primary locus of local nonspecific immune function and consists primarily of mucosa-bound lymphoid tissues. One study investigated the immunomodulatory activity of APS (139 mg/kg) of various molecular weights (157.7 × 10^3^, 69.9 × 10^3^, 22.4 × 10^3^, 13.2 × 10^3^, and 1.4 × 10^3^ Da). Via intravenous injection for 14 days to model mice, it was showed that high-molecular weight APS markedly improved the phagocytic exponent, and phagocytosis ratio in small intestine and lung, and dramatically increased the secretion of SIgA [61]. Another study confirmed that APS obviously enhanced the multiplication of mouse intestinal mucosal *γδ*T cells, enhanced the viability and cytotoxicity of APS-treated mouse intestinal mucosal *γδ*T cells [62]. Recently, scholars [63] investigated the effects of intranasal injection of APS (50 mg/kg) on mucosal immune cells in mice. APS enhanced the amount of lineage-CD11c + dendritic cells (DC) in mesenteric lymph nodes (mLN), markedly up-regulated the intracel-lular generating levels of IFN-*γ*, granzyme B, and perforin in the mLN. Besides, the levels of IFN-*γ* production by CD4^+^ and CD8^+^ T cells in mLN and lungs were also significantly up-regulated. The involvement of APS in mucosal immunity is complex and still needs to be explored.

Besides, AR has favorable immunomodulatory capacity to regulate both innate and acquired immunity. The immunomodulatory capacity of AR has been confirmed after years of experimental research. As early as 2000, it was demonstrated that APS greatly enhanced the phagocytic capacity of macrophages [64]. Afterwards, a study found that a dose-dependent increase in NK cell activity in gastric cancer rats fed with APS (100, 200, and 300 mg/kg) for 5 weeks [8]. Then, another study proved that giving APS (5,10, and 20 mg/kg) to 4 weeks old Yorkshire pigs with foot-and-mouth disease virus (FMDV) vaccine enhance the amount of CD3^+^CD4^+^CD8^+^ memory T helper cells and CD3^+^CD4-CD8^+^ cytotoxic T cells [65]. Notably, it was demonstrated that various APS with different molecular weight (Mw) (300 Da, 10 kDa, and 2000 kDa) could differently stimulate the proliferation of macrophage-occupying neutrophilic red and NK cells, and the effect of APS with 10 kDa Mw was the most significant effect [66]. Once again, it proved that the molecular weight of polysaccharides can influence immune regulation. In recent years, researchers have reported the potential mechanisms. APS has been reported to induce macrophages to express inducible nitric oxide synthase (iNOS) genes by activating the nuclear factor NF-*κ*B/Rel and decreasing the expression of the NF-*κ*B/Rel binding complex [67]. And APS was able to increase the expression of granulocyte macrophage colony-stimulating factor (GM-CSF), NO, and TNF-*α* [68]. In addition, it was found that after AR treatment, the number of ILC3 was increased, the expression of IL-17F, IL-17A, IFN-*γ*, IL-22, and GM-CSF mRNA increased as well and activation of AR improved the expression of ROR*γ*t to promote ILC3 production [69]. The above studies on the immune activity of AR showed that the immunomodulatory effects of AR developed from the influence of immune organs and immune cells.

### Hepatoprotective activity

Cholestasis is a clinical syndrome characterized by disrupted bile acid flow with no effective treatment. Chronic cholestasis can lead to liver fibrosis and cirrhosis [70]. Huangqi Decoction (HQD) consists of AR and Glycyrrhizae Radix Et Rhizoma, did good job in alleviating cholestatic liver fibrosis (CLF). A previous research reported that the addition of HQD (2 and 4 g/kg) could alleviate intestinal flora imbalance, improve gut barrier dysfunction, inhibit liver inflammation, and have a protective effect on cholestatic liver injury in cholestatic mice [71]. Another research proved its protective action on chronic cholestatic liver injury and biliary fibrosis in mice, and proposed that the mechanism may be related to the induction of Nrf2 pathway, inhibition of NF-*κ*B pathway, and improvement of bile acid (BA) stimulation of inflammation [72]. An in-depth study of the material basis and mechanism of action of HQT was reported. This study investigated the pharmacokinetics of multiple components of HQD in human plasma using ultra-high performance liquid chromatography-triple quadrupole mass spectrometry (UPLC-MS/MS), which identified 24 prototype components and 17 metabolites [73]. In addition, they found that astragaloside IV **(26)**, liquiritigenin, glycyrrhetinic acid, glycyrrhizic acid, cycloastragenol, and isoliquiritigenin could down-regulate the expression of *α*-SMA mRNA in vitro. Cycloastragenol, calycosin-7-O-*β*-D-glucoside **(68)**, liquiritin, formononetin **(64)**, glycyrrhetinic acid, and isoliquiritin down-regulated the expression of Col I mRNA. Calycosin **(67)**, liquiritigenin, isoliquiritigenin, cycloastragenol, and glycyrrhetinic acid accelerate the apoptosis of LX-2 cells. Furthermore, the antifibrotic activity of total astragaloside (AST) against liver fibrosis has been reported in some articles. Feeding cholestatic hepatic fibrosis rats with AS (14, 28 and 56 mg/kg) showed that astragaloside and cycloastragaloside alcohol significantly increased the expression of CK19 and *α*-SMA mRNAs and proteins in liver tissues [74]. There are some studies reported that mechanism of action of AS in the treatment of liver fibrosis. They proposed that AS could exert anti-hepatic fibrosis effects by inhibiting hematopoietic stem cell activation and modulating the TGF-*β*1/Smad signaling pathway [75, 76]. In addition, scholars confirmed the anti-fibrosis effect of cycloastragalus alcohol and cycloastragenol had a better antifibrotic effect at a dose of 200 mg/kg in a CCl_4_-induced hepatic fibrosis model in mice [77]. Furthermore, AR also has good effect for other liver diseases. It was discovered that feeding ApoE deficient mice with TFA (20 mg/kg), resulted in a significant reduction in total cholesterol and triglyceride levels, and it was useful for liver lipification [78]. Next, research has confirmed that AS-IV could reverse the excessive autophagy and apoptosis of hepatocytes induced by iron dextranhydride, and had a ctherapeutic effect on hepatocyte injury [79]. Additionally, APS has great potential in slowing the progression of nonalcoholic fatty liver disease (NAFL) to nonalcoholic steatohepatitis (NASH). SIRT1/PPAR*α*/FGF21, PI3K/AKT/IRS-1, AMPK/ACC, mTOR/4EBP-1/S6K1, GRP78/IRE-1/JNK, AMPK/PGC-1/NRF1, TLR4/MyD88/NF-*κ*B, and TGF-*β*/Smad are the most common mechanisms of APS [80]. These mechanisms are used to alleviate non-alcoholic NAFL by regulating lipid metabolism, endoplasmic reticulum stress (ERS), oxidative stress (OS), insulin resistance (IR), autophagy, fibrosis, inflammation, and apoptosis, respectively [80]. TCM, including AR, are characterized by multi-components and multi-targets, and a comprehensive and systematic elucidation of their mechanisms of action requires continuous exploration.

### Anti-oxidant and anti-aging activities

Aging can affect cortical neurotransmission and synaptic function, neurogenesis, vasculature, gross morphology, and cognition, and is a risk factor that cannot be ignored in our lives. While the key factor causing aging is oxidative stress, ROS, RNS balance, and redox regulation are integral to the maintenance of normal brain homeostasis [81]. AR is getting more attention due to its antioxidant activity as well as good anti-aging effects. APS was widely reported to play a beneficial effect in the modulation of aging-related diseases. Previously, APS has been proven to significantly reduce malondialdehyde (MDA) levels and increase superoxide dismutase (SOD) activity in response to d-galactose-induced senescence [82]. Furthermore, research has shown that APS attenuates hepatocyte senescence in vitro and in vivo in an H_2_O_2_-induced hepatocyte senescence model, the main mechanism of which is the reduction of reactive oxygen species levels via the AMPK/mTOR pathway, and it is noteworthy that APS is most effective at a dose of 100 μM [83]. Two other studies proved that APS prolonged the lifespan of *Drosophila melanogaster* and *Caenorhabditis elegans*. It was reported that APS reduced reactive oxygen species levels and attenuated hepatocyte senescence in vitro and in vivo via the AMPK/mTOR pathway in a model of hepatocyte senescence induced by H_2_O_2_, and APS was most effective at a dosage of 100 μM [84]. Besides, RNAi knockdown of ATF-6 prolonged the lifespan of *Caenorhabditis elegans*, demonstrating that miR-124-regulated ATF-6 contributes to lifespan extension [85]. APS might be able to modulate the miR-124 signaling pathway of ATF-6 to extend the lifespan of *Caenorhabditis elegans*.

In addition, AS and AF have been reported to exhibit antioxidant and anti-aging activities. Astragaloside and ASI **(29)** delay hydrocortisone-induced aging in rats via antioxidant and immunomodulatory effects [86]. Some scholars have investigated the mechanism of action. It was found that AS and AS-IV **(26)** can increase the antioxidant capacity of the body and eliminate senescent cells to retard the aging process by restoring the MnSOD activity and GSH/GSSG ratio and inhibite the MAPK and NF-*κ*B pathways and modulating the TGF-*β*/Smad pathway [5]. AS and AS-IV **(26)** also protected human skin fibroblasts from ultraviolet A (UVA)-induced photoaging by mechanisms related to inhibition of the MAPK and NF-*κ*B pathways, modulation of the TGF-*β*/Smad pathway, enhancement of skin cell viability under UVA irradiation, inhibition of MMP1 expression, and blocking of type I procollagen degradation [87–89]. Notably, it was confirmed that AF scavenges superoxide radicals and hydroxyl radicals, and this effect was enhanced with increasing AF concentration [90]. Isoflavones was able to prevent the decline of antioxidant enzyme activity, reduce membrane fluidity stability, chelate iron and copper ions involved in the production of free radicals, and scavenge ROS to achieve its antioxidant effect [91–93]. As the anti-aging and antioxidant activities of AR have been studied in greater depth, the development of antioxidant and anti-aging functional foods as well as cosmetic products with AR as the main ingredient has a large potential.

### Anti-inflammatory activity

Generally, AR is considered to have good anti-inflammatory activity. Over the years, scholars have proved its anti-inflammatory effect from various inflammatory models and explored its mechanism of action. For example, in lipopolysaccharide (LPS)-stimulated BMECs, APS and AS-IV **(26)** inhibited gene expression of IL-6, IL-8, and TNF-α and achieved anti-inflammatory effects by activating the Wnt signaling pathway [94]. Another study found that APS-A1 **(1)** and APS-B1 **(2)** reduced the levels of TNF-*α*, IL-6, and MCP-1 in RAW264.7 macrophages and were able to regulate NF-κB and MAPK pathways [95]. Another study found that APS inhibited the TLR4/MyD88/NF-*κ*B signaling pathway to exert anti-inflammatory effects [96]. Another study also verified this discovery. APS (200 mg/kg) can obviously decreased the level of CVB3-induced markers of cardiac inflammation (IL-1*β*, IL-6, TNF-*α*, INF-*γ*, and MCP-1), and the expression of cardiac TLR-4 and phosphorylated NF-*κ*B p65 [97]. Besides, APS increased the number of bacteria producing SCFAs, thus inhibiting the NF-*κ*B signaling pathway [98], and AR alleviated colitis symptoms in mice by regulating intestinal flora diversity [99]. Moreover, other active ingredients of AR also play a better anti-inflammatory effect. It was reported that saponins of AR inhibited excessive inflammation by interacting with a variety of inflammatory pathways, including NF-*κ*B, MAPKs, and TLR4, which was beneficial in suppressing intestinal inflammation [100]. It is worth noting that saponins of AR and APS may exert their anti-inflammatory effects via the same mechanism of action. Besides, research confirmed that AS-IV (**26**, 50 mg/kg) treatment markedly reduced in vitro IL-1*β*-induced inflammatory cytokine production in orbital fibroblasts [101]. In the latest study, researchers increased the bioavailability and BBB permeability of AS-IV by microbial and hepatic biotransformation. Through designing cell and animal experiments to observe its efficacy in a cerebral hemorrhage disease model. It was demonstrated that derivatives of AS-IV were able to significantly reduce inflammatory expression in the brain [102]. This provided new ideas for the utilization of low OB herbal components. In addition, in a mouse model of ApoE-deficient mice, TFA inhibited inflammation by decreasing miR-33 expression and suppressing NF-*κ*B activity, thereby inhibiting ABCA1/G1 activity [78]. The above evidence indicated that AR and its active components exerted anti-inflammatory effects mainly through the regulation of NF-*κ*B signaling pathway, and Wnt, MAPK, TLR4, and JNK signaling pathways [103]. JAK-STAT have also been shown to regulate inflammatory response in the body.

### Anti-viral activity

A number of studies have comprehensively demonstrated the important property of AR for antiviral activity. Enterovirus 71 (EV71) is one of the major pathogens causing severe hand-foot-and-mouth disease in children. However, there is a lack of clinically approved specific antiviral drugs for the control of EV71 infections. Study confirmed that AST can reverse nuclear atrophy and inhibit apoptosis and heat apoptosis of GES-1 cells [104]. AST deserves in-depth explore as a potential antiviral medicine for the therapy of EV71 infections. The outbreak of 2019 coronavirus disease (COVID-19), has seriously challenged the health of human beings, with a high death rate in the elderly and men [105]. As an indispensable strategy of preventing viral infections, TCM takes an essential part in epidemic defense in China. APS was proved to be an effective adjuvant for influenza and COVID-19 vaccines, mitigating the deadly attack of the influenza A virus [106]. In addition, LNPs binding AS-VII **(33)** and AS-VII **(50)** were shown to serve as effective adjuvants for in vitro inactivated H3N2 vaccines and generate cytokine responses via the Th1/Th17 pathway [107].

The efficacy of AR and its active ingredients on viral myocarditis (VM) induced by Coxsackie virus B3 (CVB3) have also been reported in many articles. For example, APS was found to have good effect in preventing myocardial injury and inflammation, which may be partially attributed to the regulation of the TLR-4/NF-*κ*B p65 signaling pathway [108]. Besides, another study found that calycosin 7-O-*β*-D-glucopyranoside (**68**, 24 mg/kg), and 14 consecutive days of in vitro treatment demonstrated its ability to significantly reduce cardiac viral titers and cardiac index [109]. Calycosin 7-O-*β*-D-glucopyranoside showed antiviral activity against CVB3-induced myocarditis both in vivo and in vitro. AS-IV (**26**, 100mg/kg) had a protective effect on cvb3-induced myocardial injury and fibrosis in mice with viral myocarditis, which may act by inhibiting the activation of the FAS/FASL signaling pathway. [110]. Furthermore, the antiviral components of AR that have been reported include anti-dengue virus activity, including astragaloside II **(35)**, astragaloside III **(27)** and astragaloside IV **(26)** [111]. Besides, APS was able to effectively prevent Muscovy duck reovirus (MDRV) infection in ducks [112], and antiviral activity against duck circovirus (DuCV) [113]. Overall, AR has antiviral activity, but its mechanism of action warrants further study.

### Anti-tumor activity

Cancer is a major reason of death and the worldwide death rate is projected to be 28.4 million cases in 2040, a 47% rise from 2020 [114]. In this paper, the mechanism of action of AR active ingredients on cancer was sorted out. Polysaccharides exhibit potent antitumor activity by inhibiting tumor metastasis and promoting immune response in a variety of cancers [115]. Studies have demonstrated that APS can inhibit lung cancer development by interfering with the S1PR1-STAT3 signaling pathway [116], as well as that APS improved the expression of CD80 and CD86, enhanced the maturation of DCs, and activated cytotoxic T-lymphocytes (CTLs) [117].

The antitumor activity of AS-IV **(26)** has been extensively reported. One study demonstrated that the bidirectional cross-talk between Nrf2/HO-1 and pSmad3C/3 L is critical in exerting the anti-hepatocellular carcinoma effect of AS-IV**(26)** [118]. Another study confirmed that AS-IV**(26)** exerts its anti-hepatocellular carcinoma effect via the same signaling pathways in C57BL/6J mouse hepatocellular carcinoma model [119]. In addition, it was observed that AS-IV**(26)** inhibited HepG2 cells through the PI3K/AKT signaling pathway and has a radiosensitizing effect on hepatocellular carcinoma cells [120]. Besides, AS-IV**(26)** can inhibited tumor growth in vivo by immune-enhancing activity by inducing CTL activity and suppressing Tregs expression [121].

The study found that TFA can decrease the expressions of IL-6 and STAT3 via the IL-6/STAT3 pathway in Lewis ruffed tumor mice, thus suppressing tumor growth. Besides, TFA had potentiating and detoxifying effects on cisplatin [122]. Calycosin **(67)** can induce cell cycle arrest and apoptosis to affect pancreatic cancer development and the signal transforming growth factor-*β* is the key to its action [123], and Calycosin through ERK/JNK pathway decreased the incidence of Abdominal Aortic Aneurysm [124]. Notably, AR protein caused programmed necrosis in HepG2 hepatocellular carcinoma cells by affecting the p53 signaling pathway [125]. This research not only provided a rationale for AR proteins against hepatocellular carcinoma, but also offered a reference for future anti-tumor studies of protein analogues. Lately, exploration of the efficacy and mechanism of AR in colorectal cancer seems to be a hot research topic. An article confirmed the inhibitory effect of AR extract on colon cancer cells MC38 and found that the inhibitory effect showed a correlation with the concentration of AR extract [126]. Another article found that AR and Curcuma aromatica Salisb inhibit colon cancer growth and liver metastasis by regulating Epithelial-mesenchymal transition via the CXCL8/CXCR2 axis and PI3K/AKT/mTOR signaling pathway [127]. Furthermore, another article investigated its mechanism of action and found that AR inhibited colon cancer development by inhibiting Wnt5/β-catenin signaling [128]. In addition to the types of cancer mentioned above, the active constituents of AR have been shown to be effective in ovarian cancer [129], laryngeal cancer [130], and Melanoma Tumor [131].

### Cardioprotective activity

A a number of cardioprotective mechanisms of the active ingredients in AR have been reported. Literatures showed that AR and its active components had significant effects on myocardial injury induced by ischemia–reperfusion and other stimuli (ischemia alone, isoproterenol, H_2_O_2_, GdCl_3_, adriamycin, and LPS). For instance, one study stabilized the hearts of isolated rats via buffer perfusion for 30 min, then via whole-brain ischemia without blood flow for 30 min, and finally via K-H buffer containing APS2-1 (**3**, 72 mg/L) and APS3-I (**4**, 62 mg/L) for 50 min and recorded heart rate, left ventricular developmental pressure (LVDP), and ventricular systolic force (± dP/dt) [12]. The results confirmed that APS significantly improved myocardial systolic and diastolic function, while reducing hypoxia/ischemia-induced release of LDH, AST, and CK. Besides, feeding AR (100–600 mg/kg) to rats with myocardial ischemia found that it reduced myocardial infarct area and lowered serum lactate dehydrogenase, creatine kinase isoform MB (CK-MB), and cardiac troponin levels [132]. A more in-depth study confirmed that feeding AS-IV (**26**, 80 mg/kg) to MI/R rats continuously for 7 days, demonstrating that AS-IV attenuates myocardial I/R injury via inhibition of CaSR/ERK1/2 and related apoptotic signaling pathways [133]. The above evidences demonstrate the role of AR mainly via the regulation of intracellular Ca^2+^ homeostasis and antioxidant activity. Myocardial fibrosis is a common pathophysiologic concomitant of many different myocardial diseases, and the mechanism of action of AS-IV for this disease has drawn the interest of researchers. It was reported that [134] found that feeding AS-IV (**26**, 10 mg/kg) for 8 days in an ISO-induced cardiac fibrosis rat model inhibited cardiac fibrosis via miR-135a-TRPM7-TGF-*β*/Smads pathway. Another study indicated that AS-IV (**26**, 40 mg/kg) decreased MI-induced myocardial fibrosis and promoted cardiac remodeling by inhibiting the ROS/Caspase-1/GSDMD signaling pathway in a mouse model of cardiac infarction [135]. Atherosclerosis is an underlying cause of coronary heart disease. In another study, feeding CVB3-induced AS-IV (**26**, 300 mg/L) to BALB/c mice for 6 months was found to reduce fibrosis in cardiac tissues, possibly due to downregulation of TGF-*β*1-Smad signaling [136]. In summary, the mechanism of AS-IV on myocardial fibrosis is through regulating TGF-*β*1-Smad, and miR-135a-TRPM7-TGF-*β*/Smads pathway. ROS/Caspase-1/GSDMD pathway were also found to be involved.

Pathological hypertrophy is recognized by systolic and diastolic hypoplasia and is a multifactorial disease. Research found that ISO-induced cardiac hypertrophy rats was fed with HQTP (80mg/kg) for 6 weeks, and showed that it could reduce ventricular wall thickness, weaken ventricular narrowing, and reduce heart weight ratio, and suggested that APS regulated TNF-*α*/PGC-1*α* signaling mediated energy biosynthesis [137]. Furthermore, research has indicated that feeding AS-IV (**26**, 20, 40, and 80 mg/kg/d) for 2 weeks could reduce the heart weight and left ventricular ratio of hypertrophic rats, dose-dependently inhibiting myocardial hypertrophy. The cardioprotective effect was associated with inhibition of TLR4/NF-*к*B signaling pathway and regulation of inflammatory factors [138]. In addition, for this pathway, APS nanoparticles alleviated sepsis-induced myocardial injury by inhibiting the TLR4/NF-*κ*B pathway [139]. The active ingredients in AR, especially AS-IV **(26)**, have shown the heart protective effects. However, due to the complexity of the mechanism of AR related active substances in treating heart disease, further pharmacological research is still needed.

### Anti-diabetic activity

Diabetes is a global metabolic disease characterized by elevated blood glucose, leading to impaired insulin secretion and damage to various tissues and organs (eyes, kidneys, nerves, heart, and blood vessels) [140]. Active substances in AR have been widely used in basic research of anti-diabetes. It has been shown that AS-IV **(26)** acts as an active anti-diabetic compound via modulation of the AMPK/SIRT1 and PI3K/AKT signaling pathways in a type 2 diabetes mouse model [141]. APS showed great potential in the therapy of diabetes and its complications. Research on its therapeutic mechanisms, it was found that APS effectively reduced fasting blood glucose levels, blood urea nitrogen, serum creatinine levels, and renal pathological damage in a rat model of diabetic nephropathy. Furthermore, APS significantly ameliorated renal injury by decreasing the expression of inflammatory factors IL-1*β*, IL-6, and MCP-1 and inhibiting the activity of TLR4/NF-*κ*B pathway in rats [142]. Recently, research indicated that APS exerts hypoglycemic effects via the intestine. They found that APS-1 significantly reduced insulin levels in type 1 diabetic (T1D) mice and improved the intestinal barrier function by regulating the expression of ZO-1, Occludin, and Claudin-1. Additionally, APS rebuilt the intestinal flora by increasing the relative abundance of *Muribaculum, Lactobacillus* and *Faecalibaculum* and inhibited the expression of pro-inflammatory factors IL-6 and TNF-*α*. They speculated that the alleviation of T1D by APS-1 may be related to the production of bacteria from short-chain fatty acids (SCFAs), which bind to GPRs and HDACs proteins and modulate the inflammatory response [143]. In a rat model of diabetic cardiomyopathy (DCM), APS attenuated cardiac hypertrophy and prevented DCM by inhibiting activation of the BMP10 pathway [144]. Besides, it was shown that APS was able to ameliorate vascular endothelial dysfunction in diabetes by stimulating macrophage polarization to M2 through enhancing the Nrf2/HO-1 pathway [145]. In addition to APS, formononontin **(64)** was found to partially attenuate mitochondrial damage and renal tubular injury in diabetic nephropathy (DN) by modulating the Sirt1/PGC-1*α* pathway in an in vivo experiment in diabetic rats [146]. Moreover, giving TFA (5, 25, and 50 mg/kg) to type 2 diabetes mellitus (T2DM) model mice for 16 weeks was able to significantly improve brain damage through modulating the gut-microbiome-brain axis. In vitro studies confirmed that TFA increased the viability of HT22 cells and preserved intestinal barrier integrity in CaCO_2_ single cell layer, and PGC1α/AMPK pathway was involved in this process [147]. Overall, APS is a potential drug candidate for the treatment of diabetes and its complications, but it still needs to be investigated in-depth studies on its mechanisms and molecular targets.

### Other activities

AR is an herb and a dietary supplement with great potential. Researchers fed APS-added diets to spotted seatrout and found that the addition of APS was effective in improving the growth and lipid metabolism of spotted seatrout, and the greatest efficacy were obtained at a concentration of APS of 0.6881 g/kg [148]. In addition, the active ingredients in AR have been reported to have antibacterial [149], hematopoietic [150], retinal protection [151], treatment of hyperlipidemia [152], and hepatoprotective effects [80].

## Clinical use

AR is one of the most commonly used medicines and is favored by doctors and patients. It has a long history of application and outstanding therapeutic effects, was named “the longest of tonic medicines” by Li Shizhen, and is widely used. This chapter describes the clinical application and efficacy of prescription or proprietary Chinese medicines containing AR.

### Immunomodulatory effect

The immunomodulatory activity of AR has been broadly investigated and the immunomodulatory effects of APS, AF, and AS in AR have been discussed in the previous review studies [156]. Clinical evidences have demonstrated the immunomodulatory effects of AR injection. To assess the effect of AR injections used with conventional therapy on chronic aplastic anemia (CAA) in a randomized controlled pilot study (n = 60). Clinical studies have found that AR injection improves T-lymphocyte subpopulations, reduces the release of negative regulatory factors such as TNF-*α* and IL-2, and promotes the immune mechanisms of hematopoiesis [157]. In addition, one clinical trial treated 100 patients with gastric cancer after major gastrectomy with AR injections in combination with conventional therapy [158]. The results showed a significant increase in CD4^+^ T-cell levels, CD4^+^/CD8^+^ T-cell ratio, IL-2 and IL-6, and a marked improvement in postoperative immunosuppression. Recently it has been reported that AR injections improved the levels of T lymphocyte subsets CD3^+^, CD4^+^ and CD4^+^/CD8^+^, and reduced symptoms in children with lupus nephritis [159], as well as the incidence of nausea and vomiting, hyperhidrosis, hemorrhage, and infections in leukemia patients following chemotherapy [160]. In addition, it was found that AR injection increased IgA, IgG, and IgM levels, improved immune function and promoted recovery in children with hand-foot-mouth disease (HFMD) [161]. AR compounds have shown potential in modulating immune function. One study was conducted on 80 patients with primary hepatocellular carcinoma surgery, who were given Huangqi Sijunzi Soup for 10 weeks, and the levels of IgG, IgM and IgA in the treatment group were significantly higher, and had better clinical efficacy [162]. Another study investigated and studied 80 patients with COPD lung and kidney qi deficiency with blood stasis, and treated them with Huangqi Danshen Yin for 8 weeks. It was found that the CD4^+^ and CD4^+^/CD8^+^ of the treatment group were higher, and the overall effective rate was higher than the control group [163]. However, the mechanism of synergism between single herbs in AR compound formula remains unclear. Currently, some scholars have investigated the synergistic mechanism of the two herbs, AR and Ginseng. It was found that AR and ginseng cooperatively regulate glycerophospholipid metabolism for immunomodulation, with pyrimidine metabolism and sphingolipid metabolism considered to be specific pathways for AR, and energy metabolism and glycerolipid metabolism specific pathways for ginseng [164]. Research on the collaborative mechanism of single drugs offered a new direction of drug development (immune enhancers) for the clinical use of Chinese medicines. However, the multi-component and multi-target characteristics of Chinese medicine compounding make the mechanism of its action on diseases very complex, so the study of its synergistic mechanism is very challenging.

### Clinical studies of AR in the heart

Clinical studies have confirmed the positive clinical efficacy of AR in many types of heart disease. Viral myocarditis (VM) is an inflammatory disease caused by a virus that damages the muscle tissue of the heart. Clinically the disease leads to acute heart failure (AHF) and sudden death with substantial complications and mortality [165]. Various active components of AR have been demonstrated to have anti-myocardial effects, including AS-IV, APS, Calycosin-7-O-beta-D-glucopyranoside, and AF. In a study, 28 randomized clinical controlled studies (2,522 participants) were evaluated clinically and preclinically in a systematic manner to assess the efficacy, safety, and possible mechanisms of AR for the treatment of viral myocarditis (VM). The data demonstrated that AR significantly reduced serum cardiac myosin and cardiac troponin I levels and improved clinical efficacy in patients with viral myocarditis [166]. Then, in a randomized, controlled, pilot study (n = 54), researchers evaluated the effects of AR injections in patients with fulminant myocarditis. The study found that surviving patients treated with AR injections had significant reductions in cardiac injury marker levels, renal function, and left ventricular ejection fraction. It was found that AR injection may be an option in situations where clinical evidence is insufficient and ambiguity remains regarding the addition of immunosuppressive agents. Meanwhile, AR injection was found effective in the treatment of viral myocarditis in a study of 120 patients with viral myocarditis who underwent AR injection for 28 consecutive days [167]. Mechanistically, AR injection inhibited miR-146b and miR-155, thus promoting immune responses, promoting the secretion of IL-10 and TNF-*β* by Treg cells and inhibiting the secretion of IL-17 and IL-21 by Th17 cells, which attenuated myocardial injury by regulating the balance of cytokine and T-cell immunity, further confirmed the immunological activity of AR injection. However, the long-term efficacy of AR injection alone is poor. It has been demonstrated that the overall efficacy of AR injection in combination with conventional therapy is significantly higher than that of conventional therapy alone. In a randomized controlled trial study (n = 70), the effect of trimetazidine combined with AR injection in treating acute viral myocarditis was evaluated [168]. Clinical studies have shown that trimetazidine, a novel cardiometabolic analog, combined with AR injection can effectively exert anti-inflammatory and anti-OFR effects in the treatment of AVMC patients. However, the mechanism of interaction between the two needs further investigation. In addition, clinical studies have shown that the clinical efficacy of edaravone, a new type of potent oxygen radical scavenger, combined with AR injection in the treatment of patients with viral myocarditis has been improved [169].

AR compounds are also effective in the treatment of heart diseases. As early as 2000, it was reported the clinical cases of HQD for the treatment of coronary artery diseases. The patients in the treatment group took HQD for 3 months, showing good efficacy [170]. Afterwards, the effects of Huangqi Baoxin Tang on cardiac function and neuroendocrine factors were further studied in clinics. After a 3-month clinical trial, it was found that its clinical efficacy may be related to the inhibition of overactivation of the RASS system, protection of endothelial cell function, and inhibition of ventricular remodeling [171]. In addition, researchers found that combining Huangqi Baoxin Tang with metoprolol could effectively improve cardiac function of patients, inhibit myocardial remodeling, and decrease the incidence of adverse drug reactions [172]. The combination of Chinese and Western medicines can not only improve the clinical efficacy, but also complement each other's advantages, which is of great research value.

### Treatment of diabetes and its complications

AR has proven to be very effective in diabetes and its complications, especially for compound formulas made from AR as the raw material. One study reported the clinical efficacy of AR injection combined with lipoic acid injection in the treatment of patients with diabetic foot by AR injection 30 mL plus saline injection to 250 ml for 28 days, the results showed an obvious reduction in the glycated hemoglobin value in the treatment group [173]. Another study reported the clinical efficacy of AR on diabetic nephropathy, with AR injection 20 mL, added to saline 250 mL intravenous infusion, once a day for 3 weeks. The total effective rate of the patients in the treatment group was 87.18%, the efficacy was obvious [174].

For the clinical efficacy of AR compounds in the treatment of diabetes and its complications, Huangqi Guizhi Wuwu Tang is very promising. Huangqi Guizhi Wuwu Tang is derived from Zhang Zhongjing's “The Essentials of the Golden Chamber”, and is a representative formula for treating “blood paralysis”. It consists of five herbs: AR, Cinnamomi Ramulus, Paeoniae Radix Rubra, Zingiberis Rhizoma Recens, and Jujubae Fructus. Currently, Huangqi Guizhi Wuwu Tang is mainly used for the treatment of hand-foot syndrome, hemiplegia in stroke, and diabetic peripheral neuropathy. The core compounds in Huangqi Guizhi Wuwu Tang include quercetin, kaempferol, catechins, and acetylcholinesterase. Through regulating blood glucose, Huangqi Guizhi Wuwu Tang can reduce insulin resistance, anti-oxidative stress, anti-inflammation, promote the release of vascular endothelial growth factor, and improve growth-induced neovascularization in vivo, which improves microcirculation, body immune regulation, regulation of lipid metabolism disorders and alleviates nerve cell ischemia and hypoxia thereby treating diabetes mellitus and its complications [175]. In a randomized, controlled, and pilot study (n = 60), the effect of Huang Qi Gui Zhi Wuwu Tang on peripheral neuropathy in type 2 diabetes was evaluated [176]. The trial proved that TCM combined with electrical stimulation was more effective than electrical stimulation alone in restoring the motor function of patients, which was favorable to the recovery of elderly patients with diabetes mellitus combined with hemiplegia of stroke. Another study also confirmed the clinical efficacy of the compound in stroke patients [177]. Furthermore, Hunagqi Gui Zhi Wuwu Tang has good clinical efficacy on diabetic foot [178] and age-related diabetic lower limb vasculopathy [179]. Overall, there is encouraging evidence that AR and Gui Zhi Wuwu Tang has better clinical efficacy in diabetes mellitus and its complications. However, the mechanism of interaction between different drugs in its prescription needs to be further explored.

### Other clinical effects

AR can also be clinically effective in the treatment of other diseases. AR injection has also been shown to be clinically effective in patients with acute respiratory distress syndrome (ARDS) [180], child with cerebral palsy [181], chronic fatigue syndrome [182], cure of neurotoxicity induced by oxaliplatin chemotherapy [183], and severe infection in the elderly [184].

**Fig. 6:**
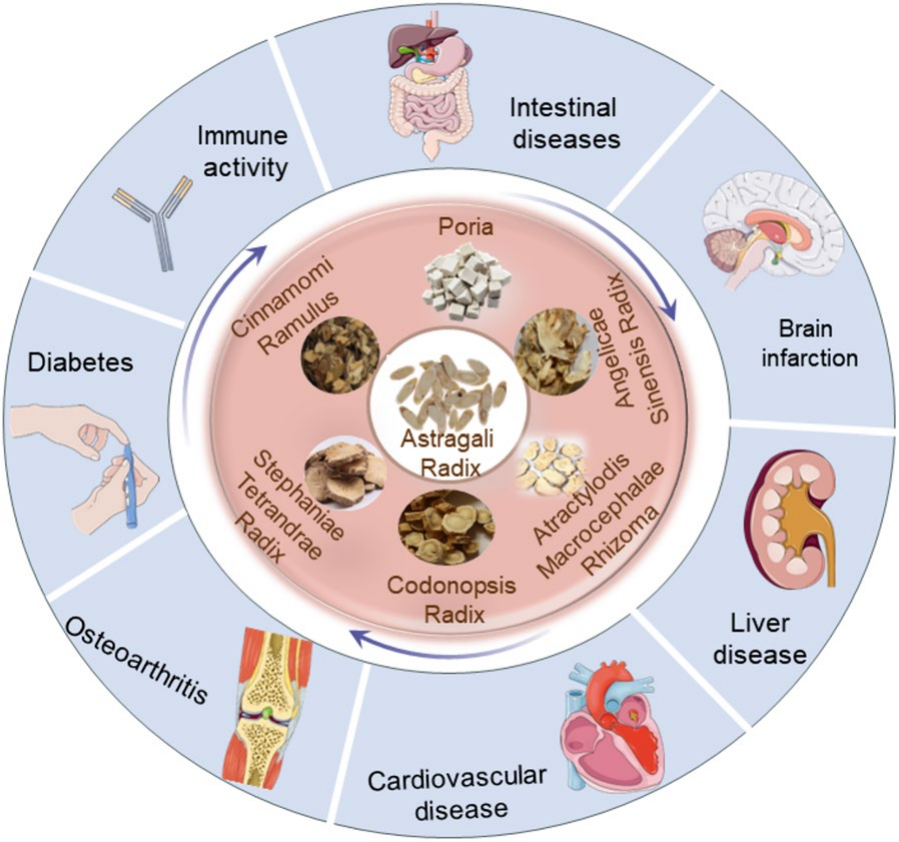
AR is used in combination with herbs to produce more diverse clinical efficacies

The AR compound has also shown promising clinical efficacy in a wide range of diseases, such as digestive disease, liver disease [185, 186], knee synovitis [187], and arteriosclerosis [188]. Intestinal diseases are common and prevalent clinical conditions. As early as 1999, scholars reported the 54 clinical cases of elephant skin and Huangqi Tang for the treatment of peptic ulcer [189]. After that, some scholars [190, 191] studied the clinical efficacy of Huangqi Tang on geriatric chronic functional constipation (FC) and found that HQD has good therapeutic efficacy on FC and low recurrence rate. Besides, it was found favorable efficacy and safety of Huangqi Jianzhong Tang herbal formula granules in the clinical treatment of chronic non-atrophic nonpneumonia [192]. It is believed that after further research on the mechanism of action of AR, there will be greater development for the clinical application of AR. The formulation of AR and the conditions treated are summarized (Table 5; Fig. 6).

**Table 5:** The clinical application of TCM formulas containing AR

Name	formula	Symptoms	Refs.
Huangqi Baoxin Decoction (黄芪保心汤)	Astragali Radix 30 g, Codonopsis Radix 12 g, Salviae Miltiorrhizae Radix Et Rhizoma 12 g, Forsythiae Fructus 12 g, Cinnamomi Ramulus 6 g, Poria 12 g, Ophiopogonis Radix 12 g, Trionycis Carapax 12 g, Schisandrae Chinensis Fructus 6 g, Angelicae Sinensis Radixi 12 g	Cardiomyopathy	[171, 193]
Fangji Huangqi Tang combined with Zhenwu Decoction (防己黄芪汤合真武汤)	Astragali Radix 30, Stephaniae Tetrandrae Radix 10 g, Atractylodis Macrocephalae Rhizoma 15 g, Glycyrrhizae Radix Et Rhizoma 10 g, Poria 30 g, Paeoniae Radix Alba 15 g, Codonopsis Radix10 g, Angelicae Sinensis Radixi 10 g, Leonuri Herba 15 g, Alismatis Rhizoma 15 g	Chronic heart failure	[194]
Huangqi Gegen Decoction (黄芪葛根汤)	Astragali Radix 20 g, Puerariae Lobatae Radix 10 g, Salviae Miltiorrhizae Radix Et Rhizoma 10 g, water 500 g	Diabetic kidney disease	[195]
Huangqi Decoction (黄芪汤)	Astragali Radix 20 g, Poria 20 g, Trichosanthis Fructus 20 g, Ophiopogonis Radix 20 g, Schisandrae Chinensis Fructus 10 g, Glycyrrhizae Radix Et Rhizoma Praeparata Cum Melle 10 g, Rehmanniae Radix 30 g	Diabetic kidney disease	[196]
Huangqi Guizhi Decoction (黄芪桂枝汤)	Astragali Radix 50 g, Paeoniae Radix Alba 20 g, Angelicae Sinensis Radixi 15 g, Cinnamomi Ramulus 15 g, Pheretima 10 g, Zingiberis Rhizoma Recens 10 g, Jujubae Fructus 10 g, Chuanxiong Rhizoma 6 g	Diabetic foot	[178]
Huangqi Guizhi Wuwu Decoction (黄芪桂枝五物汤)	Astragali Radix 30 ~ 50 g, Cinnamomi Ramulus 10 g, Paeoniae Radix Alba 15 g, Zingiberis Rhizoma Recens 10 g, Jujubae Fructus 10 g, Glycyrrhizae Radix Et Rhizoma 5 g, Salviae Miltiorrhizae Radix Et Rhizoma 15 g, Carthami Flos 5 g, Pheretima 10 g, Clematidis Radix Et Rhizoma 15 g, Angelicae Dahuricae Radix 15 g, Fruit of Fiverleaf Akebia 15 g, Gentianae Macrophyllae Radix 15 g, Notopterygii Rhizoma Et Radix 15 g	Shouder-hand syndrome	[177]
Huangqi Guizhi Wuwu Decoction (黄芪桂枝五物汤)	Astragali Radix 30 g, Cinnamomi Ramulus 10 g, Paeoniae Radix Alba 15 g, Zingiberis Rhizoma Recens 10 g, Jujubae Fructus 10 g, Trichosanthis Radix 15 g, Pseudostellariae Radix 20 g, Pheretima 10 g, Spatholobi Caulis 30 g, Salviae Miltiorrhizae Radix Et Rhizoma 20 g, Scorpio 5 g, Liquorice Tablets 5 g	Lower-extremity arterial disease (LEAD) with deficiency	[179]
Danggui Huangqi Decoction (当归黄芪汤)	Angelicae Sinensis Radixi 20 g, Astragali Radix 20 g, Codonopsis Radix 15 g, Drying Rehmannia Root 15 g, Ophiopogonis Radix 10 g, Citri Reticulatae Pericarpium 6 g, Paeoniae Radix Alba 6 g, Hirudo 3 g, Cimicifugae Rhizoma 3 g, Glycyrrhizae Radix Et Rhizoma Praeparata Cum Melle 6 g	Diabetes Foot Ulcer	[197]
Modified Bufei Huangqi Decoction (补肺黄芪汤)	Astragali Radix 25 g, Atractylodis Macrocephalae Rhizoma 20 g, Rehmanniae Radix Praeparata 20 g, Rehmanniae Radix Praeparata 20 g, Cinnamomi Cortex 20 g, Ophiopogonis Radix 20 g, Zingiberis Rhizoma 20 g, Angelicae Sinensis Radixi 10 g, Ginseng Radix Et Rhizoma 10 g, Xanthii Fructus 10 g, Schisandrae Chinensis Fructus 10 g, Achyranthis Bidentatae Radix 6 g, Citri Reticulatae Pericarpium 6 g, Glycyrrhizae Radix Et Rhizoma 3g	Diabetes Foot Ulcer	[198]
Fangji Huangqi Decoction (防己黄芪汤)	Stephaniae Tetrandrae Radix 12 g, Astragali Radix 15 g, Atractylodis Macrocephalae Rhizoma 9 g, Glycyrrhizae Radix Et Rhizoma 6 g	One lung vatilation	[199]
Huangqi Decoction (黄芪汤)	Astragali Radix 25 g, Codonopsis Radix 15 g, Cannabis Fructus 15 g, Pruni Semen 12 g, Persicae Semen 6 g, Atractylodis Macrocephalae Rhizoma 15 g, Mel 10 g, Citri Reticulatae Pericarpium 10 g	Functional constipation	[190]
Huangqi Decoction (黄芪汤)	Citri Reticulatae Pericarpium 18 g, Astragali Radix 30 g, Cannabis Fructus 20 g, Rice 10 g	Functional constipation	[191]
Huangqi Jianzhong Decoction (黄芪建中汤)	Astragali Radix 30 g, Paeoniae Radix Alba 20 g, Cinnamomi Ramulus 15g, Zingiberis Rhizoma Recens 10 g, Jujubae Fructus 4, Syrup 20 g, Codonopsis Radix 10 g, Atractylodis Macrocephalae Rhizoma 10 g, Glycyrrhizae Radix Et Rhizoma Praeparata Cum Melle 5 g	Chronic non-atrophic gastritis	[192]
Xiangpi Huangqi Decoction (象皮黄芪汤)	Elephant Hide 25 g, Astragali Radix 30 g, Atractylodis Macrocephalae Rhizoma 15 g, Paeoniae Radix Alba 15 g, Aucklandiae Radix 15 g, Jujubae Fructus 6, Glycyrrhizae Radix Et Rhizoma 10 g	Peptic ulcer	[189]
Double Flower Huangqi Decoction (双花黄芪汤)	Taraxaci Herba 30 g, Lonicerae Japonicae Flos 15 g, Forsythiae Fructus 15 g, Poria 15 g, Astragali Radix 20 g, Atractylodis Macrocephalae Rhizoma 10 g, Angelicae Sinensis Radixi 15 g, Pseudostellariae Radix 15 g, Platycodonis Radix 15 g, Angelicae Dahuricae Radix 15 g, Gleditsiae Spina 15 g	Anal Fistula	[200]
Huangqi Antidote Decoction (黄芪解毒汤)	Astragali Radix 30 g, Pseudostellariae Radix 15 g, Curcumae Radix 12 g, Atractylodis Macrocephalae Rhizoma 12 g, Cuscutae Semen 12 g, Herba Patriniae 15 g, Herba Hedyotidis 20 g, Angelicae Sinensis Radixi 12 g, Polygoni Cuspidati Rhizoma Et Radix 20 g, Rhei Radix Et Rhizoma 12 g, Salviae Miltiorrhizae Radix Et Rhizoma 15 g, Plantaginis Herba 20 g, Notoginseng Radix Et Rhizoma powder 4 g, Arecae Semen 15 g, Crataegi Fructus 15 g	Riple-negative breast cancer	[201]

## Quality control

Presently, cultivated variants and alterations of AR are flooding in the market, resulting in the poor quality of AR, and posing a challenge to the quality assurance of AR. This paper summarized the currently accepted quality evaluation standards, including the 2020 Edition of Chinese Pharmacopoeia, Twelfth Edition of Korean Pharmacopoeia, 18th edition of Japanese Pharmacopoeia Bureau, European Pharmacopoeia, Book X of "Hong Kong Standards", and Fourth Edition of Taiwan Chinese Pharmacopoeia, and gives a detailed introduction to AR from morphology, chemical information, and safety evaluation.

The 2020 edition of the Chinese Pharmacopoeia [202] specifies that genuine AR is derived from the dried root of the legume *Astragalus membranaceus* (Fisch.) Bge. var. mongholicus (Bge.) Hsiao or *Astragalus membranaceus* (Fisch.) Bge. Bge. The current market circulation of AR common adulteration includes red AR, East Russia Luo AR, pogostemon AR, and Sichuan AR.

The quality evaluation of AR starts with its traits and its microscopic identification. The Chinese Pharmacopoeia, "Hong Kong Standard", "Taiwan Standard", and "European Pharmacopoeia" are similar. Specify that AR is cylindrical in shape, measuring 30–90 cm in length and 1–3.5 cm in diameter. Its surface is light brownish yellow or light brownish in color, with irregular longitudinal wrinkles or grooves, and it is difficult to break. The fracture surface has strong fiber texture and shows powdery appearance. The bark is yellowish white, and the wood is light yellow with radial stripes and cracks. In the microscopic identification, the transverse section of AR shows multiple rows of cork cells and the phloem layer is composed of 3–5 rows of thick-walled cells. The rays on the outer side of the vascular bundle are often curved and fibers are arranged in bundles, with thick walls, woodiness or micro-woodiness, and interspersed with sieve tubes. The cambium is a ring. The xylem vessels are scattered individually or in groups of 2–3 and there are wood fibers between the vessels. Parenchyma cells contain starch grains. In addition, the Korean Pharmacopoeia and the Japanese Pharmacopoeia specifically require the absence of calcium oxalate square crystals around the fiber bundles.

The relevant regulations on the quality inspection of AR regarding moisture, total ash, heavy metals and harmful elements, and pesticide residues are shown in Table 6. The Korean Pharmacopoeia and the Japanese Pharmaceutical Bureau do not require leachate, and the fourth edition of the Taiwan Chinese Pharmacopoeia does not have the relevant regulations on moisture inspection.

**Table 6:** Quality evaluation criteria of AR in different countries and regions

Tests	2020 Edition of Chinese Pharmacopoeia	The Twelfth Edition of Korean Pharmacopoeia	The 18th edition of Japanese Pharmacopoeia Bureau	The Book X of "Hong Kong Standards"	The Fourth Edition of Taiwan Chinese Pharmacopoeia
Water content	** ≤ **10%	** ≤ **13% (Dry 6 h)	** ≤ **13% (Dry 6 h)	** ≤ **10%	
Ash	Total ash: ≤ 5.0%	Total ash: ≤ 5.0%, Acid-insoluble ash: ≤ 1.0%	Total ash: ≤ 5.0%, Acid-insoluble ash: ≤ 1.0%	Total ash: ≤ 5.0%, Acid-insoluble ash: ≤ 1.0%	Total ash: ≤ 7.0%, Acid-insoluble ash: ≤ 2.0%
Heavy metals and harmful elements	Lead ≤ 5 mg/kg; Cadmium ≤ 1 mg/kg; Arsenic ≤ 2.0 mg/kg; Mercury ≤ 0.2 mg/kg; Cu ≤ 20 mg/kg	Mercury ≤ 0.2 mg/kg; Arsenic ≤ 3 mg/kg; Lead ≤ 5 mg/kg; Cadmium ≤ 0.3 mg/kg	Heavy Metals ≤ 10 mg/kg; Arsenic ≤ 5.0 mg/kg	Lead ≤ 5 mg/kg; Cadmium no more than 1 mg/kg; Arsenic ≤ 2.0 mg/kg; Mercury ≤ 0.2 mg/kg	Arsenic ≤ 3.0 mg/kg; Cadmium ≤ 0.3 mg/kg; Mercury ≤ 0.2 mg/kg; Lead ≤ 5 mg/kg
Pesticide residues	PCNB ≤ 0.1 mg/kg	BHC, Endosulfan (sum of α,β-endosulfan and endosulfan sulfate) ≤ 0.2 × 10^–6^ respectively; Acetamiprid; Dieldrin, aldrin and endrin ≤ 0.01 × 10^–6^ respectively; DDT, Napropamide, Acetamiprid, Azoxystrobin, Triprumizole and Thiamethoxam ≤ 0.1 × 10^–6^ respectively; Imidacloprid ≤ 0.3 × 10^–6^; Phenarimol ≤ 0.5 × 10^–6^; Pimetrozine ≤ 0.05 × 10^–6^	DDT ≤ 0.2 × 10^–6^, BHC ≤ 0.2 × 10^–6^	Aldrin and Dieldrin (sum of) ≤ 0.05 mg/kg; Chlordane (sum of cis-, trans- and oxychlordane) ≤ 0.05 mg/kg; DDT ≤ 1.0 mg/kg; Endrin ≤ 0.05 mg/kg; Heptachlor (sum of heptachlor and heptachlor epoxide) ≤ 0.05 mg/kg; Hexachlorobenzene ≤ 0.1 mg/kg; Hexachlorocyclohexane isomers (α-, β- and δ- hexachlorocyclohexane) ≤ 0.3 mg/kg; Lindane (γ-hexachlorocyclohexane) ≤ 0.6 mg/kg; Quintozene (sum of quintozene, pentachloroaniline and methyl pentachlorophenyl sulphide) ≤ 1.0 mg/kg	DDT ≤ 1.0 × 10^–6^, BHC ≤ 0.9 × 10^–6^, PCNB ≤ 0.1 mg/kg
Thin-layer chromatography identification of reference materials	Radix Astragali Control Herb	Radix Astragali Control Herb	Astragaloside IV	Astragaloside II, Astragaloside IV	Radix Astragali Control Herb, Astragaloside IV
Extractives	Water-soluble extractives (cold extraction method): ≥ 17.0%			Water-soluble extractives (cold extraction method): ≥ 17.0%.; Ethanol-soluble extractives (cold extraction method): ≥ 20.0%	Water-soluble extractives: ≥ 17.0%; Ethanol-soluble extractives: ≥ 16%

Indicator components in AR that are used as quality standards are also nuanced in the currently recognized quality evaluation criteria. The 2020 edition of the Chinese Pharmacopoeia revised the content standard of AS-IV **(26)** should not be less than 0.080% by HPLC, and the content of AR isoflavone glucoside (C_22_H_22_O_10_) should not be less than 0.020% by HPLC. The Hong Kong Standard and Taiwan Pharmacopoeia only stipulate that the content of AS-IV**(26)** should not be less than 0.040%, and the eighteenth edition of Japanese Pharmacopoeia and the twelfth edition of Korean Pharmacopoeia do not have detailed provisions for the quantification of AR. Except for the pharmacopoeia-specified detection method of HPLC for AS-IV**(26)** and trichostatin isoflavone glucoside, a variety of assays coupled with HPLC have been proposed in the relevant literature, including UHPLC-MS [203], HPLC–UV [204], HPLC–DAD-MS^n^ [205], LC–ESI–MS/MS [206], HPLC-ESI/TOF–MS [207] and other liquid chromatographic methods, which are simple and exclusive. Metabolomics techniques have been widely used in the quality identification of medicinal herbs. Based on MS and NMR, metabolomics techniques can analyze metabolite types and contents [208]. Recently, researchers established a new method of reversed-phase high performance liquid chromatography-integrated pulsed amperometric detection (RP-HPLC-IPAD) for the quantitative analysis of six constituents (mauritigenin, stinging aristolochicin, and AS-IV **(26)**) in AR, which is selective, sensitive, and reproducible, and can be used directly without pre-treatment [209]. Study showed that the use of HPLC–UV-CAD was able to separate eight flavonoids and five astragalosides, which are the main chemical constituents in AR extracts [210]. This method is an easy, low-cost, and dependable chromatographic method. It was proposed that the contents of astragaloside III **(27)** and astragaloside IV **(26)** could be used as indicators for the identification of AR in Northeast, Inner Mongolia, and Shanxi. Another study also reported that the levels of chemical components in AR from different origins varied greatly, and found that AS II **(35)**, IV **(26)**, and methanone **(64)** could be used as quality indicators for quality control of AR by chromatographic fingerprinting and multicomponent quantitative analysis [206]. Furthermore, it was indicated that the content of flavonoids and saponins within AR could be determined simultaneously using HPLC–UV-ELSD method [211]. Comparison revealed that the total flavonoid content in wild AR was higher than that in cultivated AR. The evidence confirms the active ingredient AR exist difference of different regions, and with the continuous study and progress of detection methods, it provides the possibility for the more comprehensive and higher accuracy of the quality control of AR.

## Safety

AR as a medicinal herb, its safety is naturally not to be ignored. In China, according to the 2020 edition of the Chinese Pharmacopoeia, the daily dosage of AR is suggested 9–30 g. To fully explore the safety of Astragalus, many scholars have done detailed studies on the safety of AR. Since 1932, the toxic components (alkaloids, selenium, glycosides and saponins toxic components) in AR were first identified and isolated [212], and in 1965 and 1967, acute toxicity studies of AR in a chicken model were reported, revealing symptoms of paralysis and heart failure, depression, accelerated cardiac activity, and cardiac insufficiency [213, 214]. Until 1975 scholars had characterized the toxic properties of 366 species of AR, and of the 366 species, 71 were determined to be toxic. Researchers classified the toxicity of different species of AR in a study with sheep and 1-week-old chicks and found that “Class I” species (A. pterocarpus, A. convallarius, and A. diversifolius) were highly toxic to sheep at oral doses below 100 mg/kg, while “Class II” species (A. canadensis and A. cibarius) were highly toxic to sheep only at oral doses above 100 mg/kg [215]. To date, the ingredient bitartein (an indolpyridine alkaloid) contained in AR has been reported several times with acute or chronic toxicity events. In 2019, the first outbreak of AR poisoning on a farm in Argentina resulted in the death of 63 out of 70 cattle, attributed to the ingredient bitter marjoram at a concentration of about 0.096% on a dry weight basis, with symptoms of ataxia, loss of balance, and progressive wasting [216]. At the same time, studies have shown that the concentration of picloram in South American species of Astragalus spp. is greater than 0.001%, whereas the concentration of picloram in North American and Chinese samples of Astragalus spp. and Echinocereus spp. is less than 0.001%, suggesting that the production of picloram may be related to a certain type of fungus in the plant [217]. Therefore, the safety of AR should still be evaluated like any other exogenous drug before any therapeutic application.

In addition to this, this chapter compiles a survey of scholars on the safety of AR and its active ingredients based on animal models is shown in Table 7. The literature surveyed indicates that the studied AR is relatively safe at all tested levels.

**Table 7:** Summary of some toxicological studies conducted on AR

Test solution	In vivo model	Findings and key mechanisms	Novel	Refs.
Astragalosidic acid	BALB/c mice	Studies on the acute toxicity of Astragalosidic Acid (40 ~ 5,000 mg/kg for 7 days) administered orally to rats, showed no abnormal changes or deaths in rats even after a single high dose administration of 5,000 mg/kg body weight	5000 mg/kg	[222]
Cycloastragenol	Rats	The safety of cycloastragalool was assessed by repeated dosing studies (0, 40, 80, and 150 mg/kg/day) over a 13-week period with a 4-week recovery period in rats. The rat model was observed for any signs of induced toxicity by clinical observation, blood and urinalysis, and gross and microscopic pathological examination, combined with genotoxicity tests: bacterial reverse mutation test, and in vivo micronucleus test. The results showed that cycloastragalool administered to rats at 150 mg/kg/day per day was well tolerated and did not produce any toxic or genotoxic effects	150 mg/kg	[223]
Condensed fuzheng extract (sheep placenta, AR, and Polygonatum kingianum Collett and Hemsl)	Mice and rats	Acute toxicity tests confirmed NOVEL levels greater than 4,500 mg/kg by the non-death of mice in all treatments where the dose (1,125, 2,250, and 4,500 mg/kg/ d) was given continuously for more than 14 days. Sub-acute toxicity tests to detect organ damage induced by different doses (2,037, 4,075, and 8,150 mg/kg) of the substance revealed no significant changes by blood analysis and histopathological examination of the heart, liver, spleen, kidneys, thymus, testes (males) and uterus (females)	4500 mg/kg	[224]
InnoSlim (highly purified and fractionated root extracts from ARand Panax notoginseng)	Rats and mice	General toxicity was assessed by repeated dose delivery of InnoSlim (0, 400, 800, and 1,200 mg/kg/d for 28 days) to rats (females and males), and it was found that no deaths or toxic effects were observed. Genotoxicity test showed that InnoSlim had no mutagenic effect on Salmonella typhimurium and Escherichia coli. No evidence of increased micronucleus frequency due to chromosome damage in vivo has been observed in mammalian in vivo micronucleus tests	1200 mg /kg /day	[225]
HT042 (three standardized extracts from AR, *Eleutherococcus senticosus* stem, and *Phlomis umbrosa* root)	Sprague–Dawley rats	Acute and subchronic oral toxicity studies were evaluated in rats (male, female) administered HT042 orally. In the acute oral toxicity study (administration of HT042 (0, 1,250, 2,500, and 5,000 mg/kg) for 14 days), no deaths, abnormal body weight changes, or abnormal clinical signs were observed in rats Studies under subchronic oral toxicity (administration of 100 rats (640, 1,600, and 4,000 mg/kg/d) for 13 weeks, with a 4-week recovery period) had no deaths and no treatment-related clinical signs	5000 mg/kg	[226]
Myelophil (a mixture of AR and Salviae Miltiorrhizae Radix)	Beagle dogs	Acute toxicity test evaluation in 40 beagle dogs (20 males and 20 females) showed no death or drug-related clinical signs in the treatment group, except for vomiting caused by an overdose (5,000 mg/kg). Repeated toxicological tests were performed at 1250 mg/kg for 13 weeks. No drug-related abnormalities were found in histopathology, hematology, urine and biochemical analysis of rats	1250 mg/kg	[227]

Moreover, to further confirm the safety of AR, clinical data on AR were collected and analyzed in this section. According to the survey, it was found that after 114 volunteers took TA-65 on a daily dose of 5–10 mg/d (some subjects increased the dose to 25–50 mg/d after a few months), for one year, the volunteers were found to be well tolerated and had no adverse effects from the intake of TA-65 [218]. Researchers recruited 98 volunteers (38 males and 60 females) by administering Myelophil (a mixture of AR and Salviae Miltiorrhizae Radix Et Rhizoma extracts) orally (2 g per day) for 12 weeks [219]. The safety of the drug was assessed by hematological analyses. Adverse events, such as anemia, were found to occur during the experimental period, but disappeared within a short period of time and were not considered to be related to the drug treatment. There were no significant changes in biomarkers of oxidative stress and cytokines before and after treatment. A study was conducted on 80 volunteers who were given 1000 mg tablets per day containing 48% *Rubus coreanus Miquel* and AR extract [220]. No clinically significant changes in vital signs, blood and urine assays were found during the 12-week period. Notably, with the development of modern TCM, AR is used in clinical and daily healthcare applications in various preparation forms, including capsules, inhalers, drops, and injections. And the adverse reactions caused by AR injections are gradually attracting public attention. For the collected adverse events of traditional Chinese medicine injections in Hubei Province from 2014 to 2019, it was found that the total number of adverse events produced by AR injection was 502, with 53 serious adverse reactions [221]. It was positively correlated with age, the older the age, the higher the probability of serious adverse reactions. Overall, the safety of AR should be carefully evaluated after long-term clinical trials before it is used as a drug or product in clinical and daily health care.

## Product development

On November 17, 2023, AR was officially listed on the Chinese list of substances that are both food and TCM. AR is an affordable tonic. It is valuable to carry out the development of AR-related clinical drugs, foods, health care products, and cosmetics to meet health and daily necessities and other aspects of demand.

**Fig. 7:**
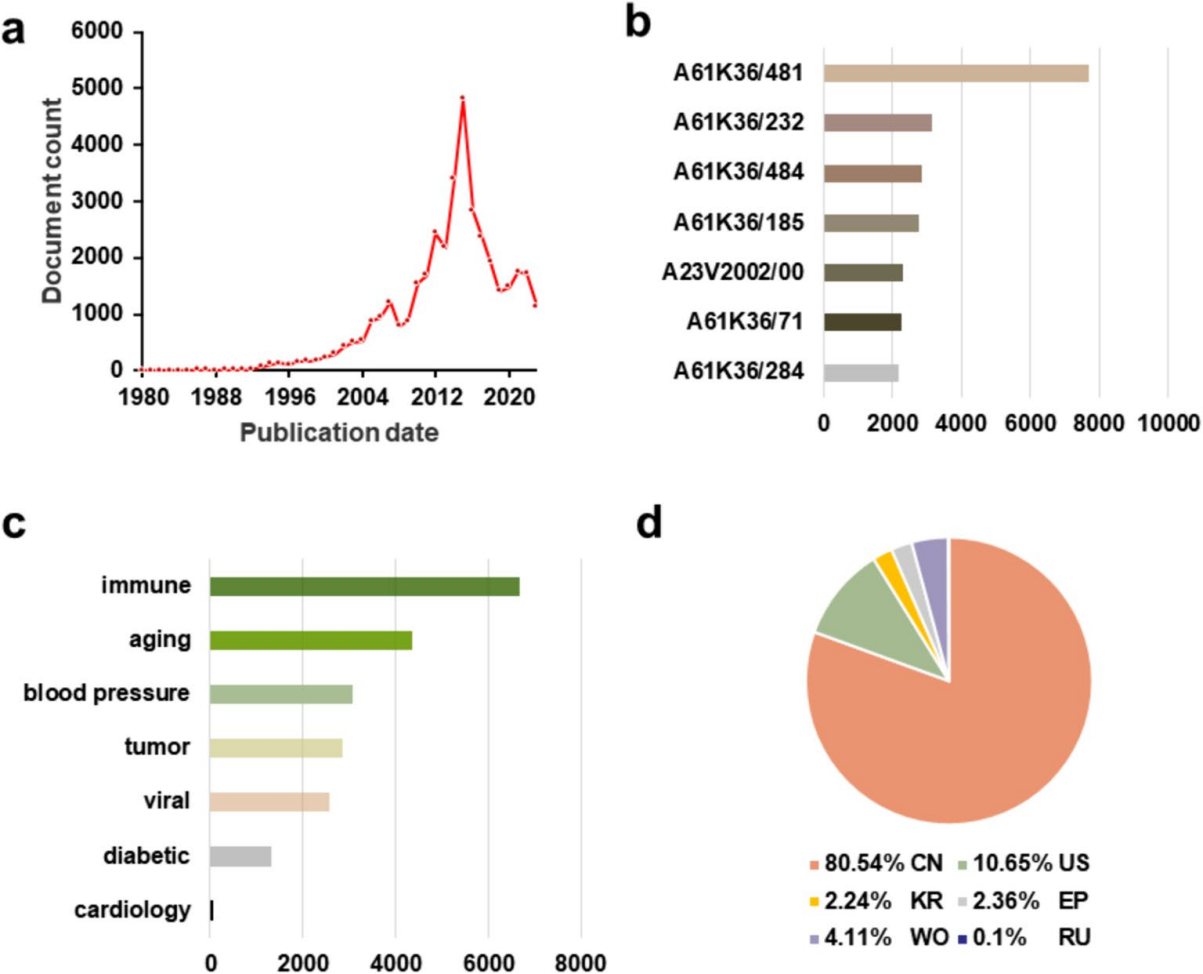
Analysis of 38,398 patents browsed on LENS.ORG (https://www.lens.org/) using search terms “Astragalus”.** a** Patent applications per year;** b** CPC classification code;** c** Effectiveness of patents.** d** Jurisdictions (CN, China; US, United States; WO, World Intellectual property organization; EP, European Patent Office; KR, South Korea; RU, Russia; JP, Japan)

AR is regarded as one of the most important qi tonic medicines and is widely used in clinical practice. At present, the number of patents of AR has reached 38398 worldwide of which China has the largest number of patents, accounting for more than 3/4 of the total. The details of the patents AR are shown in Fig. 7. In the 2020 edition of the Chinese Pharmacopoeia, the efficacy of AR-containing formulas mainly focuses on tonifying qi, followed by invigorating blood, as well as tonifying yang and the kidney. In the 2020 edition of the Chinese Pharmacopoeia, AR-based TCM formulas are Huangqi Jianwei Gao, Huangqi Shengmai Keli, and Huangqi Keli. Besides, in the 2020 edition of the Chinese Pharmacopoeia, a total of 222 AR-containing formulas were included, involving 178 prescriptions; the main dosage forms were granules (22.25%), capsules (21.62%), tablets (19.37%), and pills (17.12%). In addition, China Food and Drug Administration (CFDA) (nmpa.gov.cn) has announced 57 AR-related drugs, including compound Huangqi oral solution, Huangqi injection, Huangqi Jianzhong Pill, Huangqi Stomach Cream, Huangqi Essence, Huangqi Shengmai Granules, Xiyangshen Huangqi capsule and others. We summarized the products and effects of AR as the main component in Table 8. It is worth noting that in most EU member states, Chinese medicine granules are regarded as food supplements. Due to different policies in each country, the EU market has a huge potential waiting to be explored by the Chinese pharmaceutical industry [228]. Further studies on the mechanism of action of AR and the mechanism of pairing with other Chinese medicines will further promote the development of AR application in clinical drugs.

**Table 8:** Products developed based on AR

Drug names	Key ingredients	Main effects	Refs.
Huangqi Injection	AR extract	Cardiac insufficiency, viral myocarditis, and hepatitis with spleen deficiency and dampness	[231–233]
Huangqi granule /tablet	AR extract	Improvement of myocardial ischemia/hypoxia, enhancement of the bodyʹs immune system, and combination with various drugs such as anti-atherosclerotic, anti-inflammatory, hepatoprotective, and anti-viral drugs	[234, 235]
Huangqi Essence Oral Liquid	AR extract	For Qi deficiency and blood deficiency, limb weakness, surface deficiency and spontaneous sweating, mental deficiency or chronic illness, weak spleen and stomach	[236]
Huangqi Polysaccharides	APS	It can be used as an immune promoter or regulator, and has the functions of anti-tumor, anti-aging, anti-radiation, anti-virus, anti-oxidation, anti-stress, and so on	[68, 237]
Huangqi Polysaccharide Injection	APS	It can regulate the body's immune function and improve side effects caused by chemotherapy	[238]
Shenqi Fuzheng Injection	AR extract Ginseng Radix et Rhizoma extract	Enhance immune function and reduce chemotherapy toxicity	[239]
Compound Huangqi oral iquid	AR extract Lycii Fructus extract Cuscutae Semen extract	It is suitable for middle-aged and old people with mental fatigue caused by kidney deficiency and spleen deficiency, inappetence, insomnia and dreamless, weak waist and knee, dizziness and dizziness	[240]

With AR being officially listed on the Chinese List of Medicinal and Food Substances, it is believed that there will be more AR health products in the future. AR is safe and effective in treating diseases, and has an abundant and inexpensive source of medicine that no drug can replace. According to current research on AR, the development of AR in health care functions can be developed in terms of immune enhancement, heart health care, prevention of cardiovascular and cerebrovascular diseases, treatment and maintenance of diabetes mellitus, and anti-aging effects. With the aging of the global population, the health management for the elderly has drawn attention [229], since older people face a higher risk of disease flare-ups or drug-related side effects. The better approach to treating aging-related diseases is primary prevention [230], and health products have been developed to meet this need. Currently, there are multiple AR-based health care products, including Ba Ji Tian Huang Qi Capsules, Huang Qi Hong Jing Tian Chromium Yeast Soft Capsules, Dang Gui Ge Gen Huang Qi Capsules, Dang Gui Huang Qi Iron Tablets, and Huang Qi Dang Gui Gelatin. Its health care functions are different. Baji Tian Huangqi capsule can enhance immunity; Huangqi Hongjingtian chromium yeast soft capsule can assist in lowering blood sugar; Danggui Gegen Huangqi capsule for middle-aged and old people nourishing maintenance can enhance immunity and has a protective function against liver damage by chemicals; Danggui Huangqi iron tablets and Huangqi Danggui Agaricus are able to improve the iron-deficiency anemia. However, the dosage forms of the currently developed health products are mostly capsules, and the application of AR in health products deserves further research and development.

AR is very promising in medicinal diets. In ancient times, there are records of using AR as a medicinal diet. It is common to use AR porridge to nourish the weak body after diseases. AR is convenient to consume and can be used to stewed and boiled with meat, brewed with tea, steamed with rice and boiled with vegetables. AR is commonly used for simmering jujube, Huangqi stewed hen, and Huangqi boiled black beans are delicious, and taking these supplements regularly can make the body healthy. Yue Meizhong created "Fufang Huangqi Zhou": Coicis Semen 30 g, Vignae Semen 15 g, Galli Gigerii Endothelium Corneum 9 g, and glutinous rice 30 g. Firstly, boil 30 g Astragali Radix with 600 mL water, then add the rest of the abovementioned flavors simmering, and then take the juice into the glutinous rice boiled. After each meal porridge, chew 1 golden orange cake. Taken twice a day, it is effective in treating chronic nephritis. In addition, AR can also be cooked to function as Huangqi Danggui Wuji Tang and Huangqi Fuling Jiyu Tang. Besides, AR has been processed into a dazzling variety of products, such as Huangqi eye mask, Huangqi antibacterial liquid, Huangqi coca tea, Huangqi coffee, “precious Huangqi” drink, Huangqi wine, Huangqi moisturizing mask, Huangqi toothpaste, which have also received good feedback in the market. However, there is still a huge barrier between basic research and the industrialization of AR. With further study on the activity and mechanisms of AR and its clinical applications, it is hoped that the product development of AR can follow the step of research, and that clinical drugs and healthcare products with good efficacy and high safety can be developed, and food and various daily necessities.

## Conclusion

AR has a history of thousands of years of use in TCM. Its traditional effects are raising Yang and lifting depression, invigorating spleen and lung qi, benefiting and protecting body surface [156], blood-enriching, detoxification [241, 242] and muscle-generating [83], diuresis and detumescence, and blood-activating [150]. Known as a promising nourishing herb medicine, its major use is in the daily health care of sub-healthy persons, the elderly, and others with weak immune systems. Pharmacological studies have proven that biological activities of AR include immunomodulatory, anti-inflammatory, anti-diabetic effects, anti-viral, anti-tumor, cardioprotection, anti-hyperglycemia, anti-oxidant and anti-aging, with minor side effects. With more comprehensive research on AR and advances in production technology, AR is gradually being widely used as a clinical drug and healthcare product. Besides, the Chinese patented drugs that have been approved are reliable and clinically effective. Furthermore, development of clinical drugs and healthcare product with AR being the major ingredient, have attracted great interest from Europe, Japan, Korea, and the United States [2]. Its excellent immunomodulatory, antidiabetic and antiviral effects have been widely acknowledged. At present, there are still some problems with the relevant products, including low quality of original technology and simple processes. Therefore, with the help of health food preparation technology and modern TCM, the development of AR-related products on the basis of original preparations needs in-depth explore.

This review focused on collating the ethnomedicine uses, phytochemical and pharmacological related to immunomodulation of AR. Furthermore, the pharmacological and clinical manifestations about AR in anti-inflammation, antivirus, cardiac protection, and anti-diabetes were summarized. Besides, we summarized all the information about the compound with AR for treating diseases. Via the comparative study of related studies, we found that immunomodulatory effects are the current research trend in AR, and that this research has evolved from the initial impact on immune organs to intensive research on immune cells. It was confirmed that the components of APS, AF and AS in AR are closely associated to its immunomodulatory activity. Especially, as an important active ingredient in AR, APS has been developed as a variety of immunomodulators.

With the growth of ethnomedicine in healthcare, AR has great potential in the field of clinical drug and nutritional supplement development. But, the process of industrializing basic research is fraught with enormous challenges. Immune regulation involves massive and complex system of the whole body, but most of these previous studies are just cursory investigations of the potential mechanisms. The research of AR still needs systematic and comprehensive material basic research. Although number of identified compounds of AR have been demonstrated potent immunomodulatory effects, only polysaccharides have undergone studies on their pharmacokinetic characteristics and therapeutic mechanisms among all isolated compounds. Further in-depth investigations are required to elucidate the correlation between isolated compounds and their immunomodulatory activity, as well as to systematically explore the immunomodulatory biological effects. Significantly, the current market lacks products with AR as the main ingredient. Beyond clinical drugs, there are many possibilities for AR to be developed into products, including AR health products, AR tea, AR cosmetics. Besides, AR combined with a variety of herbs, will exhibit an enhanced therapeutic effect, including Poria, Cinnamomi Ramulus, and Stephaniae Tetrandrae Radix. Moreover, the interaction and mechanisms of action of these herbal pairs need to be further studied. In conclusion, AR is a promising tonic and can be developed into clinical drugs and nutraceuticals.

## Data Availability

Not applicable.

## Abbreviations

AR: *Astragali Radix*
AFM: Acute fulminant myocarditis
APS: Astragalus polysaccharide
AF: Astragalus flavonoids
AS: Astragaloside
AS-IV: Astragaloside IV
AST: Total astragaloside
ARDS: Acute respiratory distress syndrome
BA: Bile acid
COPD: Chronic obstructive pulmonary disease
CLF: Cholestatic liver fibrosis
CTL: Cytotoxic T lymphocyte
CaSR: Calcium-sensitive receptor
COVID-19: Coronavirus disease
CI: Cerebral infarction
CVB3: Coxsackie virus B3
CAA: Chronic aplastic anemia
CFDA: China Food and Drug Administration
CAD: Charged aerosol detector
CK-MB: Creatine kinase isoform MB
DSSD: Dampness stagnancy due to spleen deficiency
DC: Dendritic cells
DCM: Dilated cardiomyopathy
DKD: Diabetic kidney disease
DCM: Diabetic cardiomyopathy
DN: Diabetic nephropathy
DDC: 3, 5-Diethoxycarbonyl-1, 4-dihydrocollidine
EV71: Enterovirus 71
ERK1/2: Extracellular signal-regulated kinase 1/2
ERS: Endoplasmic reticulum stress
FC: Functional constipation
FMDV: Foot-and-mouth disease virus
GM-CSF: Granulocyte macrophage colony-stimulating factor
HLPC: High-performance liquid chromatography
HSC: Hepatic stellate cells
HFMD: Hand-foot-mouth disease
IR: Insulin resistance
IFN: Interferon
ISO: Isoproterenol
iNOS: Inducible nitric oxide synthase
LPS: Lipopolysaccharide
LVDP: Left ventricular developmental pressure
MLN: Mesenteric lymph nodes
MDA: Malondialdehyde
NAFL: Nonalcoholic fatty liver disease
NASH: Nonalcoholic steatohepatitis
SOD: Superoxide dismutase
SDS: Sodium dodecyl sulfate
TFA: Astragalus total flavonoids
TAS: Total Astragalus saponins
TCM: Traditional Chinese Medicine
TLR: Toll-like receptor
TLC: Thin-layer chromatography
T1D: Type 1 diabetic
T2DM: Type 2 diabetes mellitus
TFA: Flavonoids from Astragalus membranaceus
UVA: Ultraviolet A
UACR: Urinary creatinine ratio
VM: Viral myocarditis
